# Construction of Arbitrary-Order Internal Coordinate Transformations to Improve Studies of Large-Amplitude Motions

**DOI:** 10.1021/acs.jpca.6c00573

**Published:** 2026-04-28

**Authors:** Mark A. Boyer, Daniel P. Tabor

**Affiliations:** 14736 Texas A & M University, Department of Chemistry, 580 Ross St, College Station, Texas 77843, United States

## Abstract

Internal coordinates and their derivatives underpin the efficient treatment of geometry optimizations, high-resolution spectroscopic simulation, and the fitting of potential surfaces in quantum chemistry. Existing descriptions of the construction of internal coordinate derivatives generally either lack simplicity or generality. In this paper, we provide a simple framework for evaluating any internal coordinate derivative to any order and an automatic approach to obtain the corresponding inverse transformation. Through further extension to transformations between internal coordinate systems, this approach provides a complete, generic method for handling a wide variety of molecular problems. The utility of this construction is demonstrated by investigations into the behavior of internal coordinate interpolations for studying isomerizations, quantifying the coupling between carbonyl stretches and a complex stretch coordinate in an organometallic system, and analysis of the performance of a machine learned interatomic potential in computing anharmonic frequencies as a function of low-frequency coordinate distortions. This approach is shown to be numerically efficient as well as general, and a reference implementation is provided.

## Graphical abstract

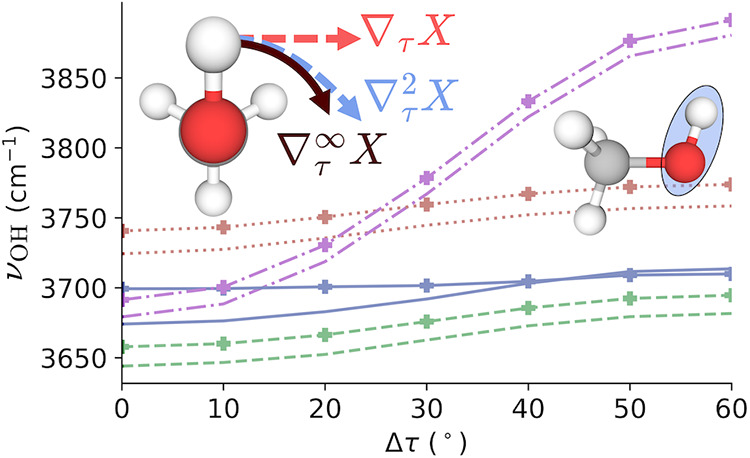

## Introduction

The use of internal coordinates in quantum chemistry is as old as the field itself. While the molecular Hamiltonian was first constructed in an external Cartesian reference frame,^1^ the appropriate transformation to a system of body-fixed internal coordinates was described shortly after^2^ and further extensions to the treatment of vibration–rotation problems developed over the following decades.^3–9^ The general approach for the construction and use of internal coordinates was popularized by Wilson,^10^ with the development of other formalisms continuing for different classes of problems.^11–16^ Internal coordinates provide a more intuitive representation for describing the fundamental physics of chemical systems,^17^ and have been extensively used to understand complicated chemical and spectroscopic problems.^18–33^

Internal coordinate approaches have also long been used to develop more efficient representations of potential energy surfaces.^34–40^ More recently, machine learning approaches have been applied to the fitting of potential surfaces for a given molecular system, with some potential for transferability.^41–44^ These models can be thousands of times faster than density functional theory and provide analytic derivatives via automatic differentiation. Studies have been performed on the spectroscopic suitability of these models, in particular, the accuracy of zero-point energies computed with these methods. An alternative to system-specific machine learned potential surfaces is the fitting of general machine learned interatomic potentials (MLIPs).^45–52^ These models have been shown to be suitable for energetic studies among other applications.^53,54^ The characterization of the performance of MLIP energies and derivatives for dynamics and spectroscopy is an open field of study.^55–57^ Internal coordinate approaches are highly applicable to such studies, providing access to intermode couplings, freezing out poorly treated motions, and allowing for efficient sampling along chemically relevant degrees of freedom.

When working in an internal coordinate system, it is generally necessary to (at least) obtain first derivatives of the internal coordinates with respect to the corresponding Cartesian coordinates of the system of interest, as these are used to construct the molecular kinetic energy operator, which is necessary to obtain vibrational wave functions, either via vibrational perturbation theory or basis set methods.^10,13,58–60^ The inverse set of derivatives, i.e., the derivatives of the Cartesian coordinates of the system with respect to the internal coordinates, are used to transform potential, dipole, and polarizability surfaces into internal coordinate representations, which allow for the evaluation of transition intensities or for the more general analysis of the potential surface, which has relevance well beyond the domain of vibrational spectroscopy or free energy calculations. Expressions for the derivatives of the most commonly used internal coordinates (*e.g.*, bond lengths, angles, and dihedral angles) with respect to the corresponding Cartesian coordinates appear throughout the literature, and higher-derivatives appear in some treatments.^10,12,14,16,61^ Similarly, numerical differentiation is very commonly used to obtain both derivatives of internal coordinates with respect to the corresponding Cartesian coordinates and Cartesian coordinates with respect to internal coordinates. With modern improvements in processing power, however, as well as efficient libraries for linear and multilinear algebraic operations, it is useful to have a simple, efficient, and general approach to the evaluation of derivatives of the most common types of internal coordinates, as well as the inverse transformations.

In this work, we detail such a general approach and provide concrete examples for how internal coordinate treatments may be used to understand complicated chemical phenomena. Specifically, the transformations described here allow for improved interpolations in internal coordinate spaces as applied to the acetylene to vinylidene isomerization process, the treatment of a low-frequency complex stretch in a cyclopentadienyl organometallic carbonyl complex and its couplings to the carbonyl stretches, and probing the behavior of machine learned interaction potentials through internal coordinate vibrational perturbation theory.

## Theory

To keep this work self-contained, we first describe the general theory of coordinate transformations, along with a simple approach to the conversion of the derivatives of any property of interest between coordinate systems. From there, we build simple expressions for the most important coordinates for chemical problems in terms of tensor/multilinear algebra expressions, along with a description of how coordinate transformations may be used to obtain expressions for any coordinate of interest. Next, we demonstrate how the inverse transformation may be obtained implicitly, with particular note of how derivatives of Cartesian coordinates embedded in an Eckart frame may be obtained solely with the corresponding derivatives of an internal coordinate with respect to a Cartesian structure. Finally, we demonstrate how more complicated coordinates and transformations between coordinate systems may be expressed in this framework, preserving differentiability.

### Theory of Coordinate Transformations

When constructing a Hamiltonian representation or expansion in internal coordinates, it is often necessary to be able to evaluate the transformation of derivatives of properties between coordinate systems in a systematic way. It is common to write these transformations down explicitly as expressions that can be evaluated in a programming language;^31,59^ however, it is also possible to represent such transformations generically. The specifics of the notation and a corresponding derivation are given in Section S2, but for convenience we will introduce notation to define the transformation of a scalar quantity *V* between coordinate systems *R* and *X* via the chain rule by

1 $$\begin{array}{ll} \nabla_{R}V & =\left[R_{\mathrm{ix}}^{\left(1\right)}V_{x}^{\left(1\right)}\right]=\nabla_{R}X\nabla_{X}V\\ \nabla_{R}^{\left(2\right)}V & =\left[R_{\mathrm{ijx}}^{\left(2\right)}V_{x}^{\left(1\right)}\right]+\left[R_{\mathrm{ix}}^{\left(1\right)}R_{\mathrm{jy}}^{\left(1\right)}V_{\mathrm{xy}}^{\left(2\right)}\right]\\ & =\nabla_{R}^{\left(2\right)}X\nabla_{X}V+\nabla_{R}X\left(\nabla_{X}^{\left(2\right)}V\right)\nabla_{R}X^{T} \end{array}$$

where ∇_*A*_^(*k*)^ *B* indicates the *k*-order tensor of derivatives of *B* with respect to the coordinates in coordinate system *A*.

With this notation, we can write down the expressions for derivative transformations up to 3^rd^ order in a compact form

2 $${\left(\nabla_{R}^{\left(3\right)}V\right)}_{\mathrm{ijk}}=\left[R_{\mathrm{ijkx}}^{\left(3\right)}V_{x}^{\left(1\right)}\right]+{\left[R_{\mathrm{ijx}}^{\left(2\right)}R_{\mathrm{ky}}^{\left(1\right)}V_{\mathrm{xy}}^{\left(2\right)}\right]}_{\left(\mathrm{ijk}+\mathrm{ikj}+\mathrm{kij}\right)}+\left[R_{\mathrm{ix}}^{\left(1\right)}R_{\mathrm{jy}}^{\left(1\right)}R_{\mathrm{kz}}^{\left(1\right)}V_{\mathrm{xyz}}^{\left(3\right)}\right]$$

where the subscripted expression (*ijk* + *ikj* + *kij*) indicates the relevant set of transpositions to ensure symmetry, as noted in Section S2. It should be noted that even though these expressions are written element-wise, they are evaluated using a series of tensor contractions and transpositions.

It is simple to see the benefits of this construction. Addition is a faster operation than multiplication, and so in the absence of hardware considerations, this expression replaces four contractions with two transposes and additions, relative to a naive recursion. Furthermore, as will be addressed later, this approach is, in general, significantly faster than the corresponding numerical differentiation. We obtain further insight into the structure of this problem by considering the exponents in each bracketed term. We can note that within each bracket, the sums of the exponents on the *R* ^(*k*)^ terms add up to the order of the derivative, and the number of *R* ^(*k*)^ terms gives the exponent of the corresponding *V* ^(*n*)^. Finally, the transpositions performed are the minimal set necessary to ensure the term remains totally symmetric, as the order of derivative operations cannot matter for physically relevant properties.

Therefore, we may write this process more generally as

$$\nabla_{R}^{\left(m\right)}V=\underset{p\in P\left(m\right)}{\sum }{\left[R_{...x_{1}}^{\left(p_{1}\right)}...R_{...x_{j}}^{\left(p_{j}\right)}V_{x_{1}...x_{j}}^{\left(j\right)}\right]}_{\left(T\left(p\right)\right)}$$ 3

where P(m) is the set of integer partitions of *m*, *j* is the length of a given partition *p*, and *T*(*p*) is the set of symmetrizing transpositions. We leave the uncontracted indices implicit in the expression above. The determination of the symmetrizing transpositions is a straightforward application of the theory of unique permutations, and is widely used in the determination of state spaces after the application of contact transformations. A derivation of the form for this work is given in Section S3.

### Coordinate Definitions and Expansions

For the problems studied in this work, we expand quantities in bond-angle-dihedral coordinates. These quantities have the following standard definitions for a given set of Cartesian coordinates *X*,

4 $$r_{\mathrm{ij}}=\left|x_{i}-x_{j}\right|$$

5 $$\theta_{\mathrm{ijk}}=\arctan2\left(\sin\left(x_{i}-x_{j},x_{k}-\mathrm{xj}\right),\cos\left(x_{i}-x_{j},x_{k}-\mathrm{xj}\right)\right)$$

6 $$\tau_{\mathrm{ijkl}}=\arctan2\left(\sin\left(n_{\mathrm{ijk}},n_{\mathrm{jkl}}\right),\cos\left(n_{\mathrm{ijk}},n_{\mathrm{jkl}}\right)\right)$$

where

$$\begin{array}{l} \sin\left(a,b\right)=\left|â\times \mathrm{b̂}\right|\\ \cos\left(a,b\right)=â\cdot \mathrm{b̂}\\ n_{\mathrm{ijk}}=x_{j}-x_{i}\times x_{k}-x_{j} \end{array}$$

It should be noted that the use of the two argument arctangent function allows for increased numerical stability as angles approach 0 and π and requires no additional logic to obtain the properly signed dihedral angle. These coordinates are displayed in Figure 1.

**Figure 1:**
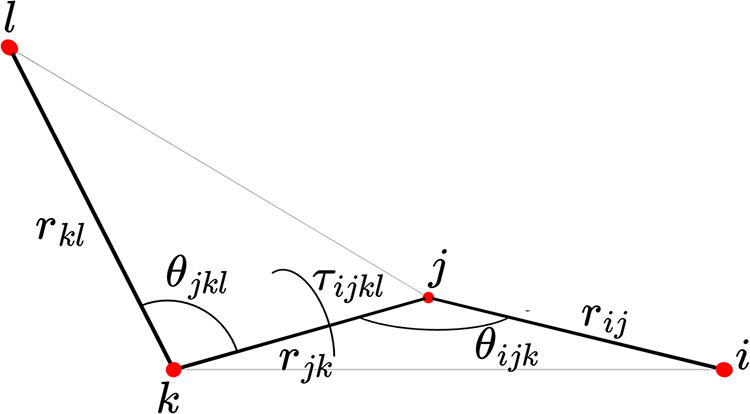
Different types of coordinates considered in this work.

To begin, we will differentiate these with respect to the components of the corresponding atomic coordinates. We begin by laying out a series of properties coming from standard multivariate calculus

7 $$\nabla_{a}\left|a\right|=\frac{a}{\left|a\right|}$$

8 $${\left(a\times b\right)}_{\gamma }=\left[a_{i}\epsilon_{i\gamma j}b_{j}\right]$$

9 $$\nabla_{X}^{\left(k\right)}\arctan2\left(\sin\theta ,\cos\theta \right)k=\nabla_{X}^{\left(k\right)}\sin\theta -\nabla_{X}^{\left(k\right)}\cos\theta$$

where ϵ is the direction cosine or 3D Levi-Cevita tensor. These expressions may be confirmed using a computer algebra system and the fact that sin^2^ θ + cos^2^ θ = 1 independent of the value of θ.

With these properties in hand, we can now expand the bond length to first order using the chain rule as

10 $$\nabla_{X}r_{\mathrm{ij}}=\nabla_{X}\left(x_{i}-x_{j}\right)\cdot \left(\frac{x_{i}-x_{j}}{\left|x_{i}-x_{j}\right|}\right)$$

This, in and of itself, is not noteworthy, but it demonstrates the general structure we will use. All internal coordinate quantities will be derived with respect to the most convenient coordinate system, and then the re-expansion procedure defined above will be used to evaluate the entire tensor.

A more noteworthy example is the first-order derivative of a bond angle, which we write as

11 $$\begin{array}{l} \nabla_{X}\theta_{\mathrm{ijk}}=\nabla_{X}\left(\sin\theta_{\mathrm{ijk}},\cos\theta_{\mathrm{ijk}}\right)\cdot \left(\cos\theta_{\mathrm{ijk}},\sin\theta_{\mathrm{ijk}}\right)\\ \nabla_{X}\cos\theta_{\mathrm{ijk}}=\nabla_{X}\left(a,b\right)\cdot \left(\frac{b}{\left|b\right|},\frac{a}{\left|a\right|}\right)\\ \nabla_{X}\sin\theta_{\mathrm{ijk}}=\nabla_{X}\left(a,b\right)\cdot \left(\frac{\left[a_{i}\epsilon_{\mathrm{ixj}}\right]}{\left[a_{i}\epsilon_{\mathrm{ixj}}b_{j}\right]},\frac{\left[\epsilon_{\mathrm{ixj}}b_{j}\right]}{\left[a_{i}\epsilon_{\mathrm{ixj}}b_{j}\right]}\right)\\ a=x_{i}-x_{j}\\ b=x_{k}-x_{j} \end{array}$$

where (*a*, *b*) indicates the concatenation of vectors *a* and *b* and It should be noted that ∇_*X*_(*a*, *b*) is sparse and sparse matrix methods should be used to evaluate that tensor product for high-dimensional systems.

While explicit formulas for these derivatives may provide geometric insight, especially at lower orders, the power of this approach is that it is straightforward to evaluate at arbitrary order, as long as one is able to evaluate ∇_*a*_^(*k*)^|*a*| for any *k*. As seen in eq 7, the derivative of the norm of a vector has a dependence on the scalar inverse of that distance, and so we end up with a growing sequence of derivatives like

12 $$\begin{array}{l} \nabla_{a}\left|a\right|=\frac{a}{\left|a\right|}\\ \nabla_{a}^{\left(2\right)}\left|a\right|=\frac{I}{\left|a\right|}-\frac{a⊗a}{{\left|a\right|}^{3}}\\ \nabla_{a}^{\left(3\right)}\left|a\right|=-\frac{{\left[I⊗a\right]}_{\mathrm{ijk}+\mathrm{ikj}+\mathrm{kij}}}{{\left|a\right|}^{3}}+\frac{a⊗a⊗a}{{\left|a\right|}^{5}} \end{array}$$

which we can see is generated by the same class of expression as in eq 3, except in place of tensor contractions, we have tensor products, and we are capped at second order in our derivative expansion. The set of symmetrizing transpositions *T*(*p*) is once again used.

With this, we have all the necessary components to construct any derivative of any internal coordinate of the form above, with the caveat that for planar dihedral angles we replace the expression in arctan with the sin of the angle between the normals. The validity of this replacement may be confirmed by numerical differentiation.

Extending this primitive set, it is common to use as coordinates for large amplitude motions some potentially nonlinear combination of bond lengths, angles, and dihedral angles. To evaluate derivatives with respect to these, it suffices to first evaluate the derivatives with respect to the initial system of bond lengths, angles, and dihedral angles, and use the prescription above to transform these derivatives into the final coordinate system. As long as the transformation from the initial internal coordinate system to the final coordinate system is differentiable, the derivatives with respect to Cartesian coordinates are straightforward to evaluate. Applications of these classes of transformations will be discussed in later sections.

### Inverse Coordinate Transformations

We have described a procedure for computing the derivatives of internal coordinates with respect to the atomic coordinates. This is all that is required to to evaluate the molecular kinetic energy operator. However, to reexpress a scalar quantity like the potential energy with respect to internal coordinates, the inverse transformation is also necessary.

For that, we will note that given coordinate systems *Q* and *R* with an invertible transformation between them, we have that for any configuration

13 $$\nabla_{R}Q\nabla_{Q}R=I$$

therefore for any *k* > 1

14 $$\nabla_{R}^{\left(k\right)}\left(\nabla_{R}Q\nabla_{Q}R\right)=0$$

Letting ∇_*Q*_^(*k*)^ *R* = *Q* ^(*k*)^ and ∇_*R*_^(*k*)^ *Q* = *R* ^(*k*)^, by expanding the relation above and isolating the term with the highest derivative in *R*, we have a series of equations

15 $$\begin{array}{l} \left[R_{\mathrm{ijx}}^{\left(2\right)}Q_{\mathrm{xq}}^{\left(1\right)}\right]=-\left[R_{\mathrm{ix}}^{\left(1\right)}R_{\mathrm{jy}}^{\left(1\right)}Q_{\mathrm{xyq}}^{\left(2\right)}\right]\\ \left[R_{\mathrm{ijkx}}^{\left(3\right)}Q_{\mathrm{xq}}^{\left(1\right)}\right]=-\left({\left[R_{\mathrm{ijx}}^{\left(2\right)}R_{\mathrm{ky}}^{\left(1\right)}Q_{\mathrm{xyq}}^{\left(2\right)}\right]}_{\left(\mathrm{ijk}+\mathrm{ikj}+\mathrm{kij}\right)}+\left[R_{\mathrm{ix}}^{\left(1\right)}R_{\mathrm{jy}}^{\left(1\right)}R_{\mathrm{kz}}^{\left(1\right)}Q_{\mathrm{xyzq}}^{\left(3\right)}\right]\right) \end{array}$$

considering now contracting along the final axis with (*Q* ^(1)^)^−1^ = *R* ^(1)^, we have

16 $$\begin{array}{l} R_{\mathrm{ijq}}^{\left(2\right)}=-\left[R_{\mathrm{ix}}^{\left(1\right)}R_{\mathrm{jy}}^{\left(1\right)}R_{\mathrm{zq}}^{\left(1\right)}Q_{\mathrm{xyz}}^{\left(2\right)}\right]\\ R_{\mathrm{ijkq}}^{\left(3\right)}=-\left({\left[R_{\mathrm{ijx}}^{\left(2\right)}R_{\mathrm{ky}}^{\left(1\right)}R_{\mathrm{zq}}^{\left(1\right)}Q_{\mathrm{xyz}}^{\left(2\right)}\right]}_{\left(\mathrm{ijk}+\mathrm{ikj}+\mathrm{kij}\right)}+\left[R_{\mathrm{ix}}^{\left(1\right)}R_{\mathrm{jy}}^{\left(1\right)}R_{\mathrm{kz}}^{\left(1\right)}R_{\mathrm{aq}}^{\left(1\right)}Q_{\mathrm{xyza}}^{\left(3\right)}\right]\right) \end{array}$$

Therefore, given an expansion in *Q* ^*k*^, we can implicitly obtain the derivatives of the inverse transformation by noting that the higher terms in the expansion of the inverse must vanish. This procedure is directly analogous to the reexpansion procedure described above, and the simplest implementation uses the same routines.

While the transformation between Cartesian coordinates and internal coordinates is not invertible, the transformation between Cartesian coordinates in the Eckart frame is.^20,62^ Therefore, by removing any component of ∇_*X*_^(*k*)^ *R* along the generators of rotation or translation,^63^ the same implicit inversion process may be used, except with ∇_*R*_ *X* obtained from the pseudoinverse of ∇_*X*_ *R*. It should be noted that the same procedure may be used to express ∇_*X*_ *R* with given ∇_*R*_ *X*, in the case that a specific set of Cartesian displacements with corresponding expansions are provided, *e.g.*, a rotation of a fragment of a system about a definite axis.

### Transforming Between Internal Coordinate Systems

It is often the case that different molecular conformations of the same set of atoms have different physically intuitive coordinate systems. To allow for the smooth transformation of properties from one set of coordinates to another, it is necessary to be able to convert between any given set of internal coordinates and another. In this work, this is done by *e.g.*, noting that in Figure 1, the side-angle-side triangle defined by *r*_*ij*_, θ_*ijk*_, and *r*_*jk*_ may be converted to the corresponding side–side–side triangle by

17 $$r_{\mathrm{ik}}=\sqrt{r_{\mathrm{ij}}^{2}+r_{\mathrm{jk}}^{2}-2r_{\mathrm{ij}}r_{\mathrm{jk}}\cos\theta_{\mathrm{ijk}}}$$

The remaining angles may be computed in a similar way. Finally, the distance *r*_*il*_ in Figure 1 may be computed from the given coordinates by

18 $$r_{\mathrm{il}}^{2}=r_{\mathrm{ij}}^{2}+r_{\mathrm{jk}}^{2}+r_{\mathrm{jk}}^{2}-2r_{\mathrm{ij}}r_{\mathrm{jk}}\cos\theta_{\mathrm{ijk}}-2r_{\mathrm{jk}}r_{\mathrm{kl}}\cos\theta_{\mathrm{jkl}}-2r_{\mathrm{ij}}r_{\mathrm{kl}}\left(\cos\tau_{\mathrm{ijkl}}\sin\theta_{\mathrm{ijk}}\sin\theta_{\mathrm{jkl}}-\cos\theta_{\mathrm{ijk}}\cos\theta_{\mathrm{jkl}}\right)$$

It is worth noting that the derivative tensors ∇_*X*_^(*n*)^ *r*_*ik*_ can be computed directly from these expressions with minimal effort using the framework described above. The direct conversion between an initial coordinate system *R* and a target coordinate system *S* may then be determined automatically via straightforward graph-based methods to determine the requisite completions.

Finally, to allow for the smooth transformation between coordinate representations, it is necessary to provide a method to interpolate between different coordinate regimes. Many interpolation schemes for molecular geometries, generally with an associated potential surface, have been proposed in the literature, each with different application domain.^64–70^ We demonstrate here a differentiable approach to interpolating between coordinate systems that can be coupled to the internal coordinate derivative and transformation schemes described above. The full mathematical description is provide in the Supporting Information, but we note that using the ability to easily convert between internal coordinate systems for different minima on a PES, we can smoothly interpolate between the coordinate systems as well. Two forms of this interpolation are provided, as sigmoidal interpolant that matches the internal coordinate behavior exactly at each minimum and an exponential form that transforms more slowly between the two regimes. Comparisons of these forms for the interpolant will be discussed further on.

## Computational Details

Electronic structure calculations were performed at four levels of theory. All coupled cluster calculations, that is CCSD­(T) optimizations and single point energies, as well as EOM-CCSD optimizations and vertical excitation energies, were performed with the Q-Chem 6.1 package using the cc-pVTZ basis.^71^ Density functional theory calculations, including optimizations, Hessian, and partial quartic force field calculations at the B3LYP and ωB97X-D3 levels of theory with the cc-pVTZ basis, as well as optimizations and Hessian calculations with the dispersion-corrected PBE functional of Martin and co-workers^72^ with the def2-TZVP basis set, were performed with the Gaussian 16 software package.^73^ Energies, gradients, and Hessians were also computed using two recently developed machine learned interatomic potentials (MLIPs) via automatic differentiation, the aimnet2_wb97m_0 model from the AIMNet2 family of MLIPs, which was trained against ∼ 10^7^ DFT calculations at the ωB97M-D3/def2-TZVP level of theory and basis set,^48^ and the extra large model from the MACE-oMOL family of MLIPs, which was trained against ∼10^8^ DFT calculations at the ωB97M/def2-TZVP level of theory.^49^ The exact model files for each MLIP are included in the supplementary data repository for this work.^74^ Throughout the rest of this work, these models will be referred to with the shorthands AIMNet2 and MACE, respectively. Optimizations were performed both with a quasi-Newton step, complemented by a simplex-based optimization using the Nelder–Mead gradient-free optimization algorithm, as gradients became insufficiently smooth for the quasi-Newton optimizers when performing constrained optimizations. Partial quartic force fields were evaluated using a step-size of 0.5 *a*_0_ for each normal mode and computing 3-point central differences on the Hessian. This step size was chosen for agreement with the third derivatives computed via automatic differentiation using both MLIPs and its use in other implementations of vibrational perturbation theory.^75^ Other finite difference schemes (5- and 7-point central differences) were also compared using a generic finite difference engine, but 3-point central differences were determined to be sufficient.^76^ Anharmonic frequencies were evaluated using the sparse matrix-based implementation of vibrational perturbation theory available in the Psience Python software package.^33,77,78^ All calculations with MLIPs were performed on an 8-core machine without a GPU.

## Results and Discussion

Before considering the direct application of internal coordinate treatments of interesting problems, it is important to discuss the benefits of the construction of internal coordinate expansions provided in this work relative to numerical differentiation. We will not compare to other direct analytic implementations of the same internal coordinates in terms of explicit expressions for the elements of the internal coordinate tensors, as the difference in performance of this construction to such approaches is likely to depend strongly on specific details of the implementation and system used. For numerical comparisons, we will consider anthracene. To obtain the derivatives of the potential and the Wilson G-matrix necessary to run second-order vibrational perturbation theory calculations, derivatives of internal coordinates with respect to reference Cartesian coordinates up to third order are required. A vectorized implementation of conversion between Cartesian and internal coordinates is used for the numerical differentiation, with a 5-point central difference stencil for the finite differences as implemented in a general-purpose finite difference library that takes advantage of the symmetries of the derivative tensors. The relative performance will differ based on implementation, but when obtaining the derivatives of internal coordinates with respect to the corresponding Cartesian coordinates, for a system with 23 atoms the approach provided in this work is ∼65 times faster and requires no code to detect when a large change in a bond angle or dihedral angle coordinate has occurred. The inverse problem of obtaining the derivatives of Eckart embedded Cartesian coordinates with respect to the corresponding internal coordinates has an even larger performance difference, with the inverse transformation described in Section 2.3 being ∼230 times faster than direct numerical differentiation while requiring no additional edge case handling. The Cartesian coordinates and *Z*-matrix used for these tests are available in the data repository supporting this work.^74^ More efficient implementations of numerical derivatives are possible with specialized or compiled implementations, but these timings are representative of a straightforward application of these approaches.

As an additional benefit, using the process of Section 2.3, it is possible to obtain the transformation coefficients to and from any set of internal coordinates by only defining the derivatives of the internal coordinates with respect to the Cartesian coordinates. This will be discussed further in Section 4.2, but allows for the construction of “delocalized” internal coordinates among other applications.^22,26,27^

### Interpolating Between Coordinate Systems

A classic example of class of chemical processes where the best internal coordinate description varies across minima is an isomerization process. Perhaps the simplest well-studied isomerization is the transformation from acetylene to vinylidene,^79–85^ shown in Figure 2.

**Figure 2:**
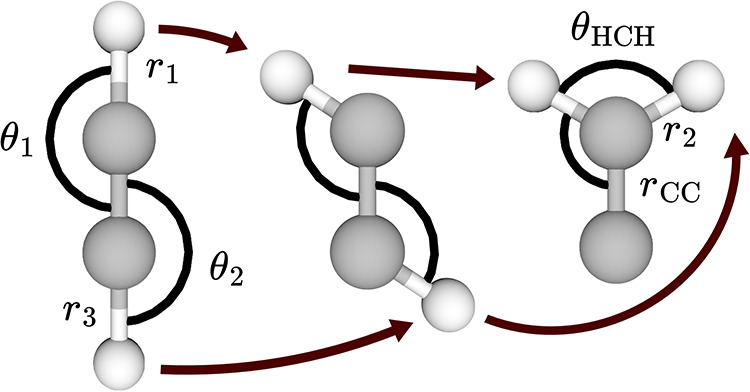
Acetylene, bent acetylene, and vinylidene with relevant coordinates displayed, as well as arrows indicating the isomerization pathway. The HCCH dihedral angle is not displayed for convenience. *r*_CC_, *r*_1_, and θ_1_ are common to both configurations, but for clarity are only displayed once.

In the acetylene-like configuration, including the bent acetylene conformer that is most stable on the *S*_1_ surface, it is natural to consider the system as two pairs of CCH angles with the corresponding distances, and a dihedral angle defining the planarity of the system. In the vinylidene-like configuration, the natural coordinates are more similar to those of formaldehyde–an HCH angle, the corresponding CH distances, a CC distance, and one of the CCH angles and the HCCH dihedral. The two CCH angles could be used in place of the HCH angle, but in both cases the key difference is that one CH distance has been substituted for another as the hydrogen shifts from being acetylene-like to vinylidene-like.

There are multiple possible interpolations that one could consider, the direct interpolation between the minima using the acetylene-like coordinates, the same but for the vinylidene-like coordinates, the two forms of interpolants discussed in Section 2.4, and a “direct” interpolation of the set of five possible CC and CH bond lengths, along with the dihedral angle to enforce planarity. The energy of the system on the ground state and *S*_1_ potential surfaces when interpolating via any of these methods is given in Figure 3. Similar plots using the AIMNet2 and MACE machine learned interatomic potentials are provided in Figure S1, where the system was reoptimized with each potential and corresponding interpolations were generated.

**Figure 3:**
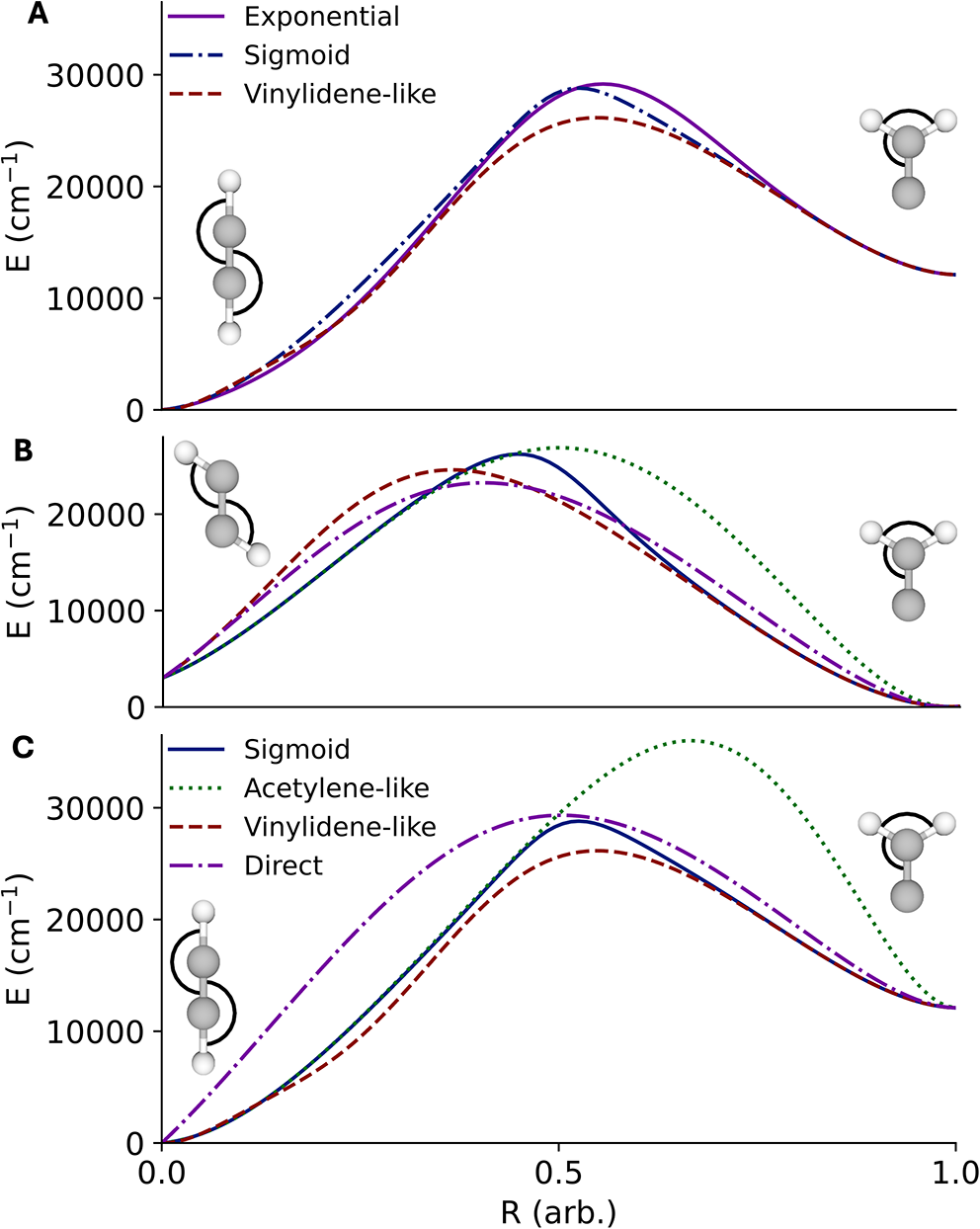
(A) Comparison of energies of the ground state at the CCSD­(T)/cc-pvTZ level of theory using geometries from interpolations using (purple, solid) the exponential form described above (blue, dot-dashed) the sigmoidal form (red, dashed) the vinylidene-like coordinates shown in Figure 2 (B) comparison of *S*_1_ energies at the EOM-CCSD/cc-pvTZ level of theory geometries from an interpolation using (blue, solid) the sigmoidal form (green, dotted) acetylene-like coordinates (red, dashed) vinylidene-like coordinates (purple, dot-dashed) the set of five CC and CH distances (C) same as (B) but using ground state energies and geometries at the CCSD­(T)/cc-pvTZ level of theory.

It is clear the most direct approach, interpolating directly based on a portion of the distance matrix, yields the least physically meaningful transitions. In the vinylidene limit, the use of vinylidene-like coordinates provides a meaningful improvement over the use of acetylene-like coordinates and as seen in Figure 3 both the exponential and sigmoidal smoothed interpolations perform similarly well. In the acetylene-limit, the picture is somewhat more complicated. For very small distortions, the acetylene-like coordinates lead to lower-energies upon distortion than the vinylidene-like coordinates, however well before the maximum in the barrier occurs this ceases to be the case. In Figure 3, it is clear the sigmoidal interpolation follows this behavior, as the geometries resembles the acetylene-like or vinylide-like interpolations at either minimum. Interestingly, the exponential interpolation consistently yields the lowest energy structures up to ∼20,000 cm^–1^. This effect is seen at both MLIP levels of theory as well.

The origin of this difference may be explored by considering the relevant interatomic distances. Considering Figure 2, there are two distances shared between the vinylidene-like and acetylene-like coordinate systems, *r*_CC_, *r*_1_, and by the fact that θ_1_ is in both systems, the corresponding third distance unlabeled in Figure 2 is implicitly defined. Therefore, in the acetylene-like interpolation, *r*_2_ may vary, while *r*_3_ is linearly interpolated, while in the vinylidene-like interpolation, the opposite is true. This may be seen clearly in Figure 4. Notably, the vinylidene-like interpolation (dashed) leads to an initial decrease in *r*_3_ (green) as the transiting hydrogen rotates out, while *r*_2_ (blue) decreases linearly. For the acetylene-like interpolation, *r*_3_ increases linearly, while *r*_2_ initially increases before decreasing. The exponential interpolation is able to sample a small increase in *r*_2_ and a small decrease in *r*_3_ as it uses information from both minima, this greater flexibility allows it to use beneficial features from both minima and hence have a lower energy interpolation. If, however, the acetylene-like coordinate system were significantly better for the acetylene-like minimum, this benefit would vanish and the sigmoidal interpolation would perform better. This is exactly what is seen for the interpolation on the *S*_1_ surface (panel B of Figure 3).

**Figure 4:**
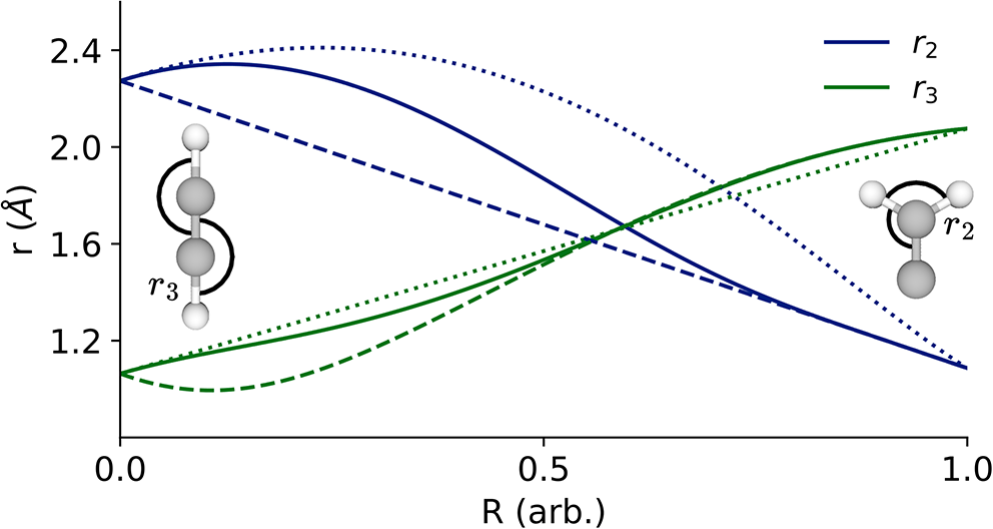
Key interatomic distances in the acetylene to vinylidene transformation, shown are *r*_2_ and *r*_3_ as labeled on the figure and in Figure 2. These distances are plotted for the exponential interpolation as described in Section 2.4 (solid), the vinylidene-like interpolation (dashed) and the acetylene-like interpolation (dotted). The corresponding energies may be see in Figure 3.

### Generalized Internal Coordinates and Vibrational Couplings for an Organometallic Complex

It is often the case that a coordinate of interest cannot easily be included in a nonredundant set of internal coordinates. To illustrate this, we will consider the cyclopentadienylmolybdenum tricarbonyl ([CpMo­(CO)_3_]^−1^) complex anion. The neutral dimer of this complex has been studied previously,^86–88^ and cycylopentadienyl-metal complexes are of general interest in inorganic synthesis.^89–93^ The important modes of [CpMo­(CO)_3_]^−1^ for this analysis are shown in Figure S2, including the cyclopentadienyl torsion and the complex stretch, which changes the distance between the cyclopentadienyl moiety and the MoCO_3_ group without changing the relative orientation. The idealized version of this coordinate is depicted in Figure 5. The spectroscopy of metal carboynl compounds is well-studied, and the primary carriers of intensity in these systems are the carbonyl stretches.^94–97^ Therefore, it is potentially of interest to consider how the position of the cyclopentadienyl group modulates the carbonyl stretch frequencies as a reporter on the coupling between these motions.

**Figure 5:**
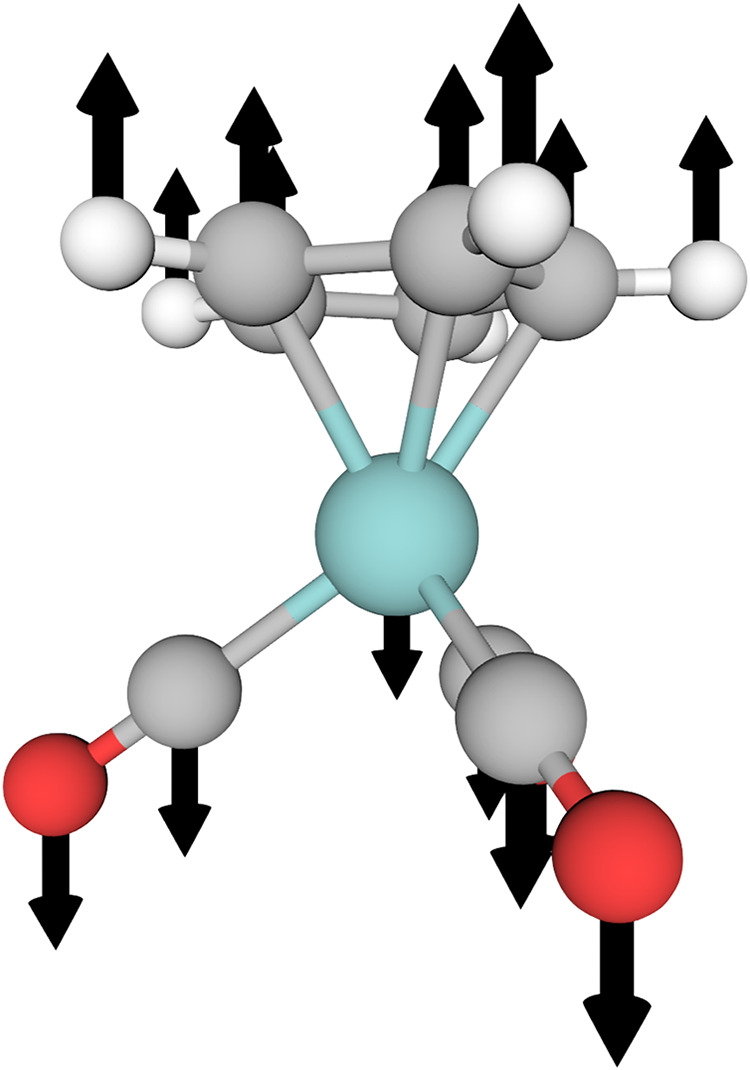
An idealized coordinate for the complex stretch in [CpMo­(CO)_3_]^−1^, defined by displacing along the axis connecting the centroid of the cyclopentadienyl moiety and the Mo atom.

We start by considering a relaxed scan about the torsional coordinate. All electronic structure calculations for this complex were done using the dispersion-corrected PBE functional of Martin and co-workers^72^ with the def2-TZVP basis set and an effective core potential on the molybdenum atom as recommended by consensus models of Kulik and co-workers.^98–100^ The Gaussian 16 ^73^ package was used for these electronic structure calculations. The geometry was optimized, and the torsion coordinate was scanned in 5 increments of 18° to cover a period of 72°, with the remaining coordinates allowed to relax at each step. Over this range the magnitude of the frequency of the torsional coordinate spans a range of 2 to 8 cm^–1^, suggesting that the cyclopentadienyl moiety rotates freely with negligible impact on the CO stretch frequencies. While such a low-frequency coordinate is particularly well suited to internal coordinate studies, the lack of definite coupling prevents definite conclusions from being drawn. We will therefore consider the complex stretch, defined for the purposes of this study as the distance between the centroid of the cyclopentadienyl carbons and the molybdenum atom. This coordinate has an equilibrium value of 2.0645 Å, and we will consider displacements of 0.1 Å, scanning from 1.8645–2.3645 Å. Concretely, in the relaxation process, a dummy atom was placed at the cyclopentadienyl centroid and a *Z*-matrix was constrained to ensure the cyclopentadienyl centroid position was preserved. Across this scan, the nominal complex stretch mode varies from 396 to 148 cm^–1^ while the antisymmetric CO stretches vary from 1844 to 1831 cm^–1^ and the symmetry CO stretch goes from 1940 to 1951 cm^–1^. This is plotted in Figure 6. This implies a moderately sized coupling between these coordinates.

**Figure 6:**
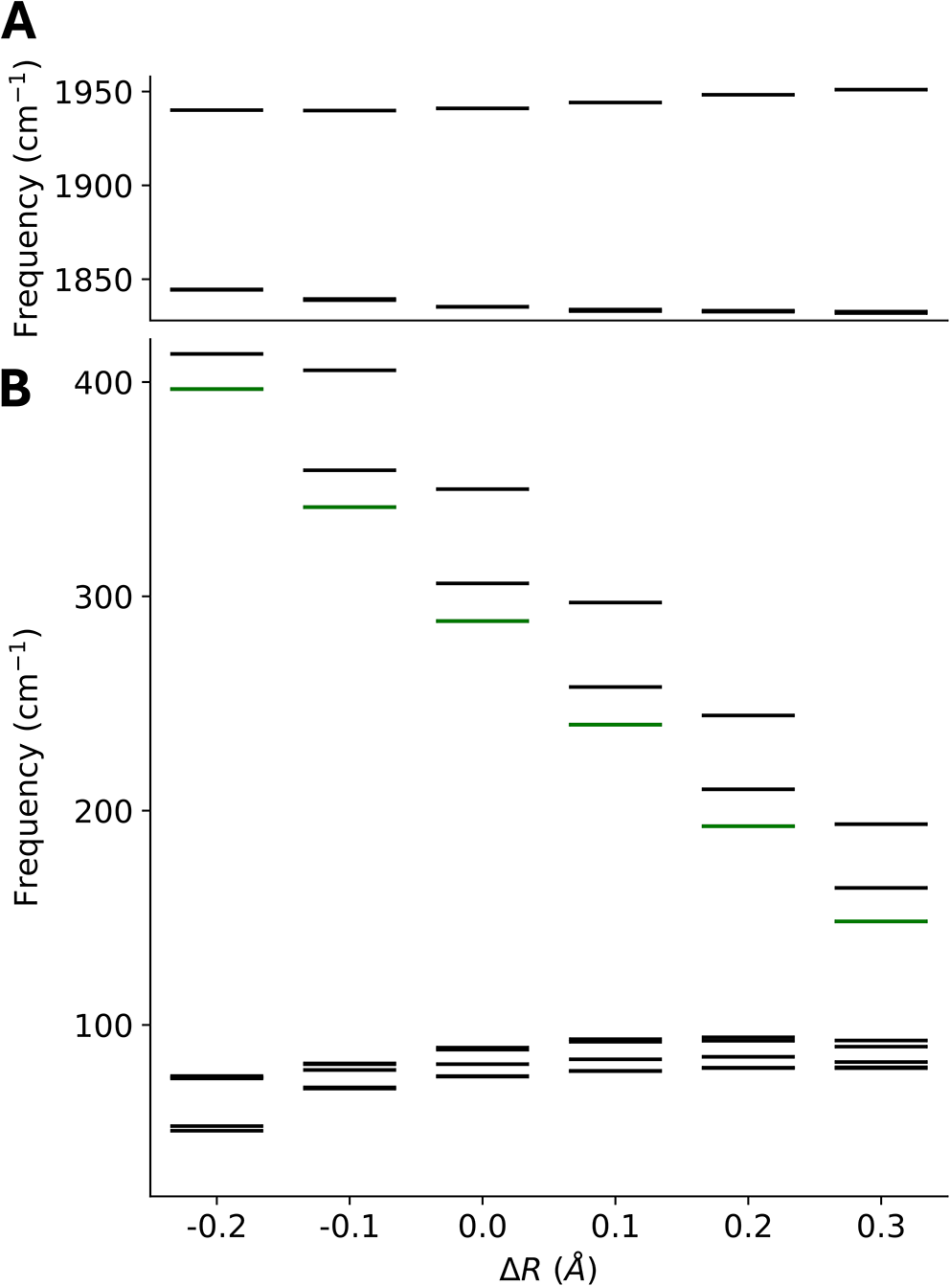
Variation of the normal-mode frequencies of [CpMo­(CO)_3_]^−1^ as a function of the complex stretch coordinates. (A) the variation of the CO stretch modes (B) the variation of the low-frequency modes with the nominal complex stretch mode in green.

However, the nominal complex stretch also includes the MoCO bend and CO torsions, as can be seen in Figure S2, which will in turn affect the coupling. To account for that, we would like to construct a pure complex stretch coordinate and a set of local CO stretches at each point along this scan so that we can see how the bilinear couplings between these coordinates evolve as a function of the centroid-Mo distance. This could be done via the addition of a dummy atom and further constraints, but the methods described in this work provide for a more direct construction. We will approach this in two distinct ways. In the first we will make use of the symmetry of the system. To construct the complex stretch mode, we will note that it is the coordinate that symmetrically increases or decreases the CMo distances for the carbons in the cyclopentadienyl moiety, while leaving the CC and CH bond lengths unchanged and not modifying any of the Euler angles between the cyclopentadienyl and MoCO_3_ moieties. Therefore, given an initial set of internal coordinates provided as *Z*-matrix for the system, we can construct a redundant set of coordinates by adding in all of the CMo, CC and CH distances. The coordinate of interest is then the following symmetric combination:

19 $$S=\frac{1}{\sqrt{5}}\left(r_{1}+r_{2}+r_{3}+r_{4}+r_{5}\right)$$

where *r*_*i*_ is one of the C–Mo distances. We then find any four linear combinations of the five {*r*_*i*_} orthogonal to this coordinate to define a new set of internal coordinates. This new set of internal coordinates, {*S*_*i*_} is a linear transformation of the initial set of internal coordinates {*R*_*i*_}, and the derivative tensors ∇_*X*_^(*n*)^ *S* may be evaluated using the transformations described above, noting that ∇_*R*_^(*n*)^ *S* vanishes for *n* > 1. In general, however, this set of coordinates will be overcomplete and so it is necessary to reduce them down to a space of 3*N* – 6 coordinates. This is done in two steps, first a set of delocalized internal coordinates is constructed in the standard way.^26^ Next, this set of 3*N* – 6 is relocalized using a least-squares transformation to make the delocalized coordinate transformation matrix look maximally like the identity matrix. In this construction, we ensure the stretch coordinate defined in eq 19 and the CO stretch coordinates undergo no mixing. After these transformations, we have a set of 3*N* – 6 coordinates, *Q*, and derivatives ∇_*X*_^(*n*)^ *Q* that can be used to assess the coupling between the CO stretches and the complex stretch coordinate *S*. For convenience and generality, in the implementation of the procedure described above, the internal coordinates for the Cp-Mo subsystem were fully symmetrized by embedding the set of symmetry operations for a *C*_5*v*_ point group in the frame of the subsystem and reducing as appropriate,^10^ but in general any set of coordinates including *S* and the full space of CC and CH stretches will work.

An alternate approach to the procedure described above is more direct, but requires more specific knowledge of the coordinate of interest. In this, we note that the coordinate we would like to describe, pictured in Figure 5, involves displacements of the atoms along the axis connecting the centroid of the cyclopentadienyl and the Mo atom. We can therefore find a linear combination of the internal coordinates of the system that at linear order maximally look like this mode. Specifically, we take an initial set of internal coordinates that comprise a *Z*-matrix for this system and find a linear combination of the derivatives ∇_*R*_ *X* that maximally agrees with the displacement vector of interest using least-squares. This procedure is best applied in mass-weighted coordinates. We then construct 3*N* – 7 linear combinations of internal coordinates orthogonal to this coordinate to obtain a complete coordinate system. This approach is analogous to the expressing translation-rotation internal coordinates in terms of primitive internals.^101^

The results of this analysis are provided in Table 1. Comparing the complex stretch frequencies, we note that the specific coordinate treatment has a large apparent effect on the associated local mode frequency, with the direct construction consistently giving higher frequency modes, particularly as the Cp-Mo distance shrinks, giving a local frequency of 596 cm^–1^ using symmetry coordinates and 867 cm^–1^ using local coordinates, relative to a corresponding nominal Cp-Mo stretch normal mode of 397 cm^–1^. This difference is driven by subtle differences in the treatments of the coordinates, and is minimized when applying a pure scaling to ensure the local modes are equivalent to the normal modes of the system, up to a unitary transformation.^102^ The rescaled frequency comparisons, along with corresponding bilinear coupling elements, are provided in Table 2.

**Table 1:** Local Stretch Frequencies in [CpMo­(CO)_3_]^−1^ Compared to Normal Mode Frequencies^a^

	Normal			Symm.		Direct	
Δ*S*	S	CO_*a*_	CO_*s*_	S	CO	S	CO
–0.2	397	1844	1940	596	1869	867	1869
–0.1	342	1839	1940	486	1863	687	1863
0.0	288	1835	1941	393	1859	541	1859
0.1	240	1833	1944	320	1858	427	1858
0.2	193	1833	1948	248	1858	319	1858
0.3	148	1832	1951	196	1858	229	1858

a Frequencies (in cm^–1^) of the complex stretch mode S and the local carbonyl stretches CO, antisymmetric carbonyl stretch CO_*a*_ and symmetric carbonyl stretch CO_*s*_ at different amounts of displacement along the complex stretch in Å.

**Table 2:** Local Stretch Frequencies and Bilinear Couplings in [CpMo­(CO)_3_]^−1^ After Scaling^a^

	Symmetrized			Direct		
Δ*S*	S	CO	w	S	CO	w
–0.2	443	1756	–30	473	1756	31
–0.1	372	1749	–29	395	1749	–29
0.0	312	1744	–26	325	1744	–25
0.1	257	1741	–22	266	1741	22
0.2	205	1740	–19	208	1740	–18
0.3	156	1739	–15	151	1739	15

a Frequencies (in cm^–1^) of the complex stretch mode, S, the local carbonyl stretches, CO, and the corresponding bilinear coupling, *w*, at different amounts of displacement (Δ*S*, in Å) along the complex stretch after a pure scaling transformation to make the coordinates equivalent to the normal modes up to a rotation.

After rescaling, the discrepancy between the symmetrically constructed and directly constructed complex stretches decreases and the trend is consistent with the nominal mode. The rescaled CO stretches have notably lower local mode frequencies than the CO stretch normal modes. This is driven by strong couplings of a CO stretch to the corresponding MoCO bend, and the normal-mode frequencies are recovered when diagonalizing within the subspace of CO stretches and MoCO bends. As the Cp-Mo distance increases, the coupling between the CO stretch and the complex stretch coordinate decreases, along with the frequency. This result is not particularly surprising, but no additional calculation or approximation was needed and similar analyses may be performed efficiently on more complicated systems using the approach detailed above. This approach also shows how different coordinate systems may lead to different interpretations of spectroscopic observables and how to ameliorate some of these differences.

### Understanding the Performance of Machine Interatomic Learned Potentials Using Internal Coordinate Perturbation Theory

As a final application of the utility of the internal coordinate transformations detailed above, we will analyze how machine-learned interatomic potentials perform in computing anharmonic frequencies for methanol using nearly degenerate vibrational perturbation theory. Methanol provides a useful test system for probing MLIP performance with respect to anharmonicities, as it is a simple, covalently bound organic molecule that contains an isolated anharmonic OH stretch, a set of nearly degenerate CH stretches, and there is high-quality benchmark data to support further study, particularly the vibration–rotation coupling in the torsional degree of freedom.^103–109^

In Table 3, we provide the anharmonically corrected OH stretch, CH stretch, HCH bend, and HOCH torsion frequencies for methanol evaluated using nearly degenerate second-order vibrational perturbation theory with quartic force fields at the B3LYP and ωB97X-D3 levels of theory using the cc-pVTZ basis set, as well as the AIMNet2 machine-learned interatomic potential, with details of the model given above.^48^ The corresponding mode vectors are depicted as Modes 1–7 and 12 in Figure S3. B3LYP was used due to literature precedent^110^ and the AIMNet2 potential was trained against electronic structure calculations at the ωB97M-D3 level of theory, hence the inclusion of both levels of theory. Calculations were performed with a quartic force-field in Cartesian displacement coordinates (labeled Cart. in Table 3) and an internal coordinate expansion (labeled Int.). The Cartesian coordinates and *Z*-matrices for these calculations as well as the full inputs and outputs from anharmonic calculations are provided in the supplemental data repository for this work.^74^ In the absence of resonance handling, the choice of coordinate system does not impact the final frequencies and intensities as may be seen in Table S1,^59,111^ however when accounting for the resonances in the system differences appear. Initially, one should note that ωB97X-D3 and B3LYP stretch and bend frequencies differ between treatments by at most 6 cm^–1^, while the AIMNet2 results may differ by as much as 70 cm^–1^. It must be kept in mind that AIMNet2 was not trained against anharmonic data and so this result appears simply to indicate that more training is required to obtain reliable anharmonic results. While this is true, the picture is more complicated, as seen in the treatment of the torsion. At the harmonic level, all three treatments provide a torsional frequency greater than 200 cm^–1^, with B3LYP and ωB97X-D3 providing frequencies of 291 and 298 cm^–1^ respectively, while AIMNet2 gives a frequency of 209 cm^–1^. Once anharmonic corrections are included, this frequency at the ωB97X-D3 level drops to ∼12 cm^–1^ indicating that this mode for the system is not treated well even at the level of theory against which AIMNet2 was trained with. It is noteworthy that the internal coordinate expansion provides more reliable results at the AIMNet2 level, relative to ωB97X-D3, due to decreased coupling of the torsion to other coordinates, but the results at either level of theory appear unreliable.

**Table 3:** Harmonic and Anharmonic Frequency Comparisons for Methanol Across Levels of Theory and Coordinate Systems^a^

	B3LYP/cc-PVTZ			ωB97X-D3/cc-pVTZ			AIMNet2/ωB97M-D3		
States	Harm.	Cart.	Int.	Harm.	Cart.	Int.	Harm.	Cart.	Int.
OH	3829	3643	3647	3918	3708	3708	3917	3603	3675
CH	3108	2985	2980	3134	2991	2985	3135	2909	2962
CH	3040	2875	2879	3067	2868	2870	3076	2979	2979
CH	2994	2813	2818	3013	2809	2812	3030	2868	2911
HCH	1509	1466	1466	1516	1474	1474	1513	1430	1524
HCH	1498	1458	1458	1506	1471	1471	1480	1533	1533
HCH	1477	1446	1446	1488	1455	1455	1473	1495	1497
HCCH	291	222	223	298	11	12	206	276	73

a Comparison of harmonic (Harm.) and anharmonic frequencies in cm^–1^ computed with nearly degenerate vibrational perturbation theory using expansions of the Hamiltonian in Cartesian displacement coordinates (Cart.) and internal coordinates (Int.) with optimized geometries and partial quartic expansions computed at the B3LPY/cc-pVTZ and ωB97X-D3/cc-pVTZ levels of theory/basis and the AIMNet2 machine learned potential trained against ωB97M-D3 calculations. Deviations between Cartesian and internal results are driven by strong couplings in the perturbation theory that are handled variationally.^77^

To address this issue with the treatment of the torsional degree of freedom, we will make further use of the internal coordinate expansions and, applying techniques derived from reaction path normal-mode analysis,^63^ project the torsional degree of freedom out of the expansions at all levels of theory. Operationally, this is performed by expressing the normal modes as expansions in internal coordinates and then removing the component along the torsional degree of freedom to construct a set of 3N-7 torsion-projected modes. These modes are then used to evaluate the anharmonic frequencies as before. In order to gain more insight into exactly how the MLIP compares to the DFT levels of theory, we will extend this analysis to geometries beyond the equilibrium torsion angle. To do so, we first fix the torsion angle at some distortion from its equilibrium value and optimize in the remaining degrees of freedom, once again making use of the efficient internal coordinate transformations detailed above to transform the Cartesian coordinate gradients. Having done so, we apply the same reaction path projection as before to obtain modes with no component along the torsional degree of freedom and perform the vibrational perturbation theory calculations in this subspace. Similar approaches have been used to obtain VPT2 energies at saddle points.^112^ The results of this analysis for the AIMNet2 MLIP at distortion angles of 0°, 30°, and 60° are provided in Table 4 and results for all levels of theory are provided in Tables S2 and S3. Here, even upon distortion, we see that the differences between the Cartesian and internal coordinate treatments remain small, and so for all further results only the internal coordinate result will be reported. The improvement in agreement between the Cartesian and internal expansions in the absence of the torsion is an example of the overestimation of couplings between modes in the Cartesian representation and an example of how to circumvent these overestimates.

**Table 4:** Harmonic and Anharmonic Frequency Comparisons for Methanol Across Distortion Angles and Coordinate Systems^a^

	0°			30°			60°		
States	Harm.	Cart.	Int.	Harm.	Cart.	Int.	Harm.	Cart.	Int.
OH	3917	3661	3672	3913	3692	3691	3906	3714	3712
CH	3135	2968	2968	3121	2974	2974	3096	2994	2994
CH	3075	2974	2973	3077	2971	2970	3089	2970	2970
CH	3030	2879	2889	3026	2880	2886	3030	2870	2880

a Harmonic and anharmonic stretch frequencies for methanol evaluated in the same way as in Table 3 using the AIMNet2 machine learned interatomic potential, but with the HCCH torsional coordinate projected out at distortions along the torsional coordinate of 0, 30, and 60° from equilibrium.

To have a more complete comparison, we extend this analysis to the full range of symmetrically distinct distortions. In Figure 7, the deperturbed frequency of the *anti*-CH stretch is plotted as a function of distortion angle. Deperturbed frequencies are used to provide more useful comparisons across levels of theory.^33,58,59,75,113^ To provide a richer comparison in the space of MLIPs, we have included the MACE-oMOL family of potentials in this analysis. In terms of performance, constrained optimizations and partial quartic force field evaluations were ∼10 times slower with MACE than AIMNet2, but this is notably on an 8-core machine without a GPU. Both approaches are likely to be dramatically faster when GPU acceleration is available. We see clearly that while both DFT levels of theory have a decrease in this frequency, the AIMNet2 stretch increases, with the effect increasing with distortion angle. MACE, by contrast, has a decrease in the frequency *anti*-CH stretch, but of a much larger magnitude than either DFT level of theory. All levels of theory have the character of this mode smoothly change from pure *anti*-CH stretch in the equilibrium structure to the antisymmetric CH stretch of the pair of *anti*-CH stretches in the eclipsed structure.

**Figure 7:**
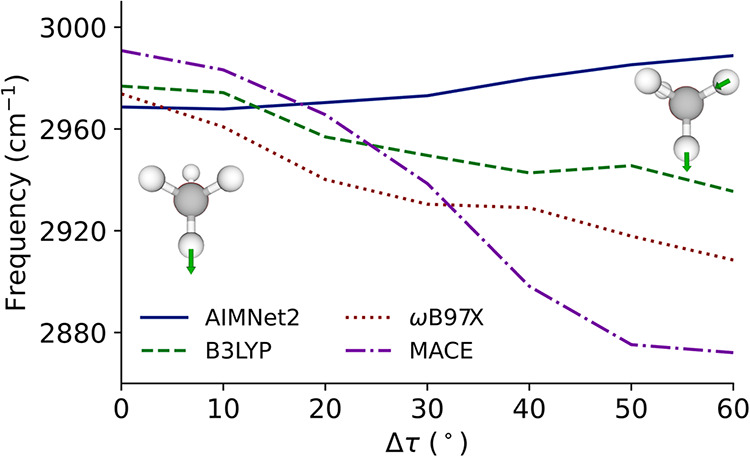
Anharmonic frequency of the *anti*-CH stretch in methanol evaluated at different distortion angles.

It becomes interesting, then, to assess which modes behave similarly between the DFT levels of theory and the MLIP treatment and what the origins of these differences are. We will begin by extending the CH stretch discussion. In Figure 8, all three CH stretch frequencies are plotted for the different electronic structure treatments across different distortion angles. While for the two DFT levels of theory the ν_2_ and ν_3_ are well separated in frequency, using AIMNet2 these frequencies are quite similar and using MACE the splittings are significantly overestimated. At the harmonic level, all three approaches are in general agreement, as seen in Figure S4, but by evaluating the anharmonic frequencies in a restricted mode space, it is possible to get insight into the origins of the trends in the anharmonic frequencies.

**Figure 8:**
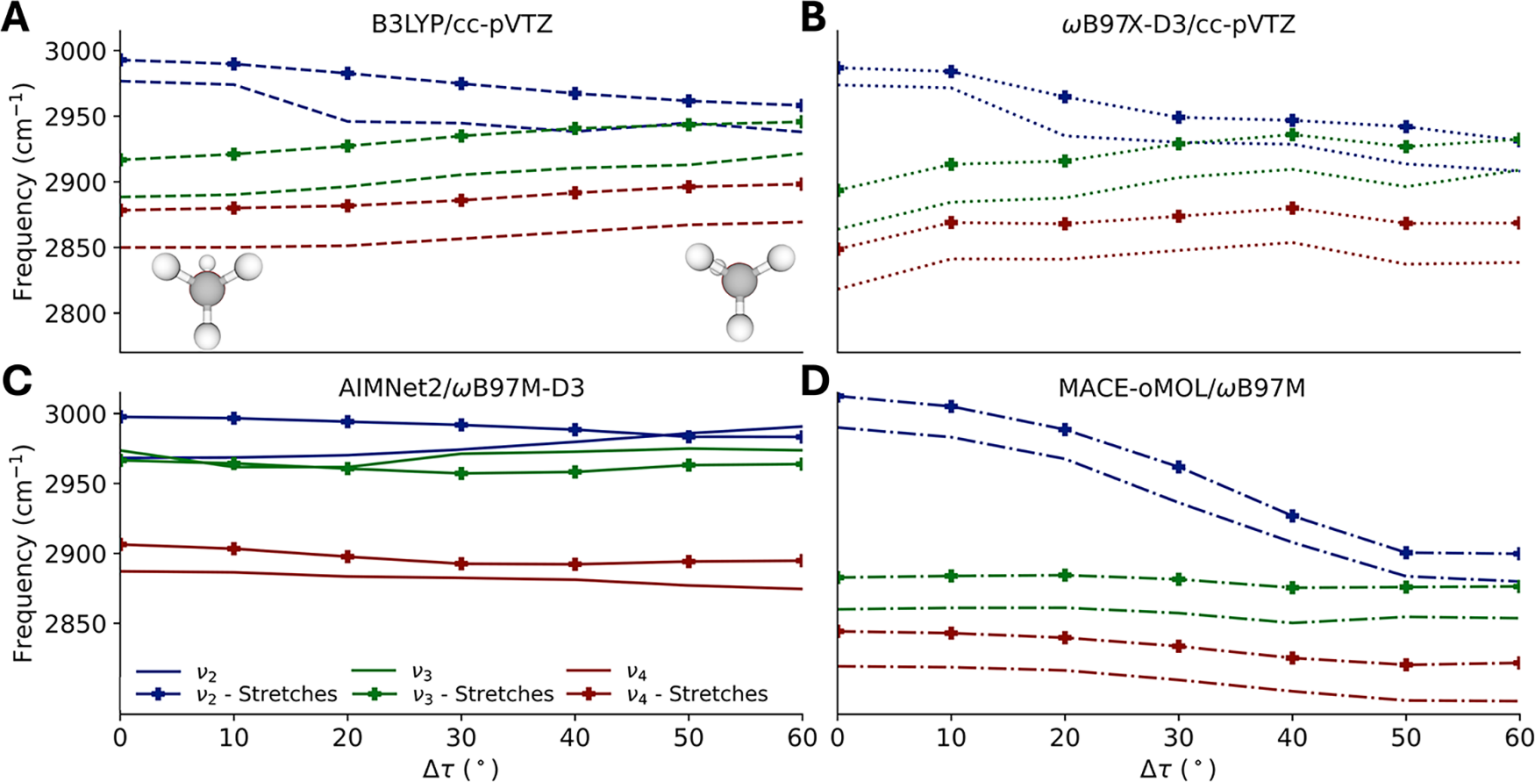
Comparison of the three CH stretching frequencies across distortion angles and levels of theory. Energies, gradients, and Hessians are evaluated with (A) DFT at the B3LYP/cc-pVTZ level of theory/basis (B) at the ωB97X-D3/cc-pVTZ level of theory/basis (C) the AIMNet2 machine learned interatomic potential with specific model given in the text (D) the MACE-OMOL interatomic potential with specific model given in the text. Unmarked curves are evaluated in the full space of reaction path modes. Markers indicate the corresponding values evaluated in the subspace of the CH and OH stretches.

In the lines with markers in Figure 8, the CH stretch frequencies are plotted as a function of distortion angle with perturbation theory corrections evaluated in a mode space only containing the OH and CH stretches. By doing this, we are able to remove errors in the coupling of the stretches to the bends and other lower-frequency degrees of freedom when evaluating anharmonic corrections with the MLIPs. Notably across levels of theory, neither ν_3_ nor ν_4_ are strongly affected by subspace restrictions, in terms of a qualitative change in shape, indicating the corrections to these modes mostly arise from diagonal anharmonicities and coupling to the other stretches. By contrast, ν_2_ is strongly affected for AIMNet2, increasing in frequency when all modes are included, but decreasing in frequency in either subspace. For either DFT level of theory or MACE, we see the subspace restrictions having relatively minor effects on the changes in frequency upon distortion, adding a roughly constant increase in frequency, or corresponding constant decrease in anharmonicity. Interestingly, the AIMNet2 CH stretch frequencies when evaluated within the stretch subspace are qualitatively in good agreement with the DFT levels of theory with respect to the splittings between the stretches. This suggests that using internal coordinate transformations to restrict modes to linear combinations of stretches and high-frequency bends might provide a route to obtaining reasonable anharmonic corrections from machine-learned interatomic potentials.

Moving then to the remaining stretch degree of freedom, in Figure 9 the OH stretch frequency is plotted as a function of distortion angle across levels of theory and reaction path mode spaces. Here, surprisingly, the anharmonic OH stretch frequency for AIMNet2 is in qualitatively good agreement with the B3LYP OH stretch frequencies when all modes are included in the subspace, while this agreement degrades with further restriction. For MACE, the same overestimation of changes in anharmonicity is seen as with the CH stretches.

**Figure 9:**
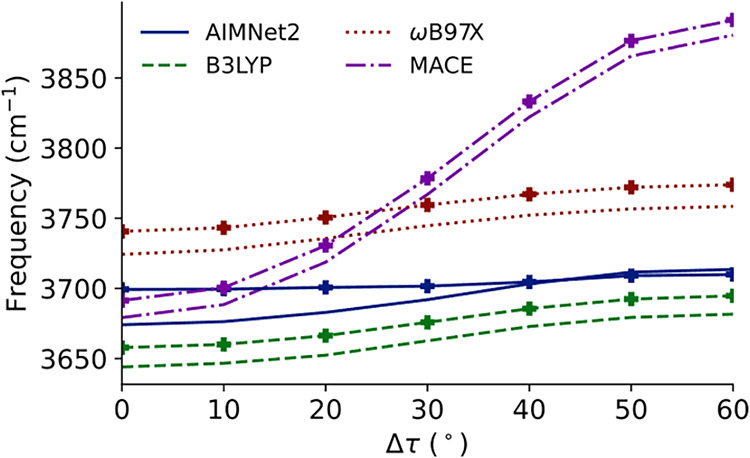
Anharmonic frequency of the OH stretch in methanol evaluated at different distortion angles and levels of theory. Markers indicate the corresponding values evaluated in the subspace of the CH and OH stretches.

Unlike the CH stretches, even at the harmonic level the OH stretch disagrees substantially with the DFT levels of theory, as shown in Figure S5. The apparent agreement at the anharmonic level is then an overcorrection of the harmonic frequencies. This is clear when considering the pure anharmonic contribution to the OH stretch, where for either DFT level of theory the effect of the mode space restriction is to act as a constant shift on the anharmonic corrections, while for the AIMNet2 potential the anharmonic corrections change dramatically with greater subspace restriction. This is demonstrated in Figure S7.

In particular, this suggests that AIMNet2 results have coupled the OH stretch too strongly to the other modes of the system, this is in spite of the fact that the OH stretch mode has no mixing to the other internal coordinates of the system, as can be seen when expressing the modes as expansions in internal coordinates. This effect persists when using third derivatives from automatic differentiation, suggesting this is not an artifact of numerical differentiation. This is potentially connected to the disagreement between the DFT and MLIP levels of theory at the harmonic level. The qualitative disagreement between the anharmonic corrections is still minimized with restriction to the stretch mode subspace, suggesting that using harmonic frequencies from a higher level of theory with cubic derivatives (potentially scaled) at the MLIP level provides a potential route to efficient anharmonic corrections.^114^

## Conclusion

We have demonstrated how a simple combinatoric approach to the construction of internal coordinate derivatives can be implemented and applied. This approach couples easily with further coordinate transformations, and a generic algorithm for handling such coordinate transformations has been provided. The derivatives constructed in this manner are numerically stable and are much more computationally efficient than the corresponding numerical differentiation and scale more favorably with system dimension.

The differentiable conversion between coordinate systems allows for richer interpolations in internal coordinate space and the ability to express complicated Cartesian space motions as simple, potentially symmetry-adapted, functions of internal coordinates simplifies the exploration of vibrational couplings between modes in noncovalently bound complexes. Finally, the efficient construction of high order derivatives of coordinate transformations enables the use of vibrational perturbation theory in a reaction path formalism along low-frequency coordinates. Such calculations provide a strong test of the performance of modern machine-learned interatomic potentials (MLIPs), which are generally trained to at most reproduce harmonic frequencies. While the anharmonic corrections obtained along the scanned coordinate using an MLIP may deviate significantly from those obtained via density functional theory, by reducing the space of coupled modes to include only high-frequency modes, this deviation may be minimized. By restricting the coordinate space used to construct the modes of the system, this deviation may potentially be further minimized with less approximation.

It should be noted that the approaches detailed in this work do not fully address the core issue of coordinate system choice in internal coordinate treatments. Redundant coordinate treatments, as have been mentioned, are one possible solution to this issues.^26,29^ Localized normal modes may provide another, which may be supplemented by an internal coordinate system.^115–119^ The efficient evaluation of derivatives provides a route to the optimal choice of internal coordinates for a given problem in a given configuration, and the coordinate system interconversions provided here may allow for the connection of locally optimal coordinate systems.

Similarly, the comparisons between MLIPs and DFT in this work reflect only the performance of two state-of-the-art machine learned interatomic potentials at the given moment. As further training is performed and anharmonic training data is incorporated, such comparisons will need to be updated. As a final caveat, the form of the interpolation provided in this work is just one of many possible choices, and interpolations that make use of energetic information or directly sample a reaction path will likely exhibit much better performance.

In other contexts, we have used this coordinate construction to perform *ab initio* molecular dynamics simulations in internal coordinates and understand the appearance of spectral features in multidimensional spectroscopies of large systems. A numerically efficient implementation using tensor operations is available,^76,78^ and a repository containing all code and data used in this work is available as well.^74^ The approach detailed above may easily be extended to coordinates beyond bond lengths, angles, and dihedral angles, and various types of implicitly defined coordinates like out-of-plane wags and in plane rocking motions have been implemented as well. Derivatives and coordinates computed in this way may be integrated into other packages, particularly for the evaluation of properties beyond second order.^120–122^ Further efficiency may be obtained by GPU acceleration of the tensor operations.

## Supplementary Material



## Data Availability

The data that support the findings of this study are available within the article and its Supporting Information. The code and data used to produce the results in this work are provided in the Zenodo repository (https://doi.org/10.5281/zenodo.18202625).

## Notes

Discussion of the key theory behind coordinate transformations; a construction of symmetrizing transpositions; mathematical background on multiple coordinate system interpolating forms; interpolation energies for the acetylene to vinylidene isomerization using MLIPs; selected normal modes of [CpMo­(CO)_3_]^−1^; normal modes of methanol; anharmonic frequencies of methanol; anharmonic frequencies of methanol using MLIPs; anharmonic frequencies of methanol using DFT; harmonic frequency comparisons for the CH stretches in methanol; harmonic frequency comparisons for the OH stretch in methanol; anharmonic frequency comparisons for the OH stretch in methanol across subspaces; and the pure anharmonic correction in methanol across subspaces (PDF)
