# Supplementary material for: Construction of Arbitrary-Order Internal Coordinate Transformations to Improve Studies of Large-Amplitude Motions
Source: J Phys Chem A. 2026 Apr 28;130(18):3680–94. doi: 10.1021/acs.jpca.6c00573 (PMC13158920; doi:10.1021/acs.jpca.6c00573)
Supplement: Supplementary file 1 [file jp6c00573_si_001.pdf]

Supporting Information for A Construction of Arbitrary Order Internal Coordinate Transformations to Improve Studies of Large Amplitude Motions

Mark A. Boyer[1, a)] and Daniel P. Tabor[1, b)]

Texas A & M University, Department of Chemistry 580 Ross St College Station, TX 77843, United States of America

[a)] Electronic mail: maboyer@tamu.edu

[b)] Electronic mail: daniel_tabor@tamu.edu

CONTENTS

S1. Theory of Coordinate Transformations S3

S2. Symmetrizing Transpositions S5

S3. Interpolating Between Coordinate Systems S7

S4. Interpolation Energies using MLIPs S9

S5. Normal Modes of Cyclopentadienyl Molybdenumtricarbonyl S10

S6. Normal Modes of Methanol S11

S7. Table of Anharmonic Frequencies S12

S8. Table of Anharmonic Frequencies using MLIP Levels of Theory S13

S9. Table of Anharmonic Frequencies using DFT Levels of Theory S14

S10. Harmonic Frequency Comparison of CH Stretches in Methanol S15

S11. Harmonic Frequency Comparison of the OH Stretch in Methanol S16

S12. Anharmonic Frequency Comparison of the OH Stretch in Methanol S17

S13. Pure Anharmonicity of the OH Stretch in Methanol S18

S1. THEORY OF COORDINATE TRANSFORMATIONS

A linear transformation between coordinate systems, R and X, for example, is commonly used and is given by

$$R(\Delta X; X_0) = R_0 + \nabla_X R \Delta \tag{S1}$$

where X_0 is the reference geometry expressed in the coordinate system X, R_0 is the same geometry expressed in the coordinate system R, $\nabla_X R$ is the matrix of first derivatives of the coordinates in R with respect to those in X evaluated at X_0, and X is a displacement vector in coordinate space X. The Duchinsky transformation used in the computation of Franck-Condon factors is an example of this class of coordinate transformation.[1] More generally, this can be expressed as a Taylor series up to order n

$$R(X; X_0) = R_0 + \sum_{k=1}^{n} \frac{\nabla_{\mathcal{X}}^{(k)} \mathcal{R}}{k!} \odot^k \Delta X \tag{S2}$$

where $\nabla_X^{(k)} R$ is the kth order tensor of derivatives of the target coordinates R with respect to the base coordinates X and $\nabla_X^{(k)} R \odot^k \Delta X$ represents the iterated contraction of the displacement vector ΔX with the corresponding axes in the derivative tensor. This is sometimes referred to as a total contraction.

Any scalar quantity, V, expanded in the base coordinate system X may then be re-expanded in the target system R through the multivariate chain and product rules. The first-order and second-order contributions are straightforward to evaluate, and are

$$\nabla_R V = \nabla_R X \nabla_X V \tag{S3}$$

$$\nabla_R^{(2)} V = \nabla_R^{(2)} X \nabla_X V + \nabla_R X \left(\nabla_X^{(2)} V \right) \nabla_R X^{\intercal} \tag{S4}$$

At third order and above, the use of matrix algebra ceases to be practical, as a large sequence of transpositions is required to obtain the correct evaluation order. For this work, we use an adapted form of Einstein notation in which we will let

$$R^{(k)} = \nabla_R^{(k)} X$$

$$V^{(k)} = \nabla_X^{(k)} V$$

$$[R^{(k_1)}_{i_1 \dots i_n} V^{(k_2)}_{i_1 \dots i_n}] = \sum_{i_1 \dots i_n} (\nabla_R^{(k_1)} X)_{i_1 \dots i_n} (\nabla_R^{(k_2)} X)_{i_1 \dots i_n}$$

$$[A^{(k)}_{i_1 i_2 i_3}]_{i_2 i_3 i_1 + i_3 i_2 i_1} = \text{transpose}(A, (i_2 i_3 i_1)) + \text{transpose}(A, (i_3 i_2 i_1))$$

that is, we let base coordinate system be implicit, and terms in brackets are contractions along the shared indices. It should be noted that one should not directly evaluate summations of this form, as efficient routines for such contractions exist in all modern programming languages. The final piece of notation is simply a compact way to represent sequences of transpositions. Finally, we will assume that all indices for derivatives come first, then indices for quantities, i.e. $R^{(k)}$ will have k indices representing the choice of target coordinates R and the final index will be for the base coordinates X.

S2. SYMMETRIZING TRANSPOSITIONS

The only difficulty in this transformation is the determination of the set of permutations $T(p)$. For this, we note that the number of transpositions for a partition p of m is a multinomial coefficient of the form

$$N(T(p)) = \frac{m!}{p_1!...p_j!c_1!...c_k!} \tag{S5}$$

where c_1 to c_k are the counts of the unique entries in p, i.e. for $p = (3, 3, 2)$, $c = (2, 1)$. From this alone, instead of evaluating $T(p)$ we could simply use every valid permutation and normalize by the number of expected permutations. This will be inefficient, however, as the number of excess additions grows factorially. We can make this more efficient by working with unique permutations. To motivate this, we will consider that in the 3rd order case the relevant transpositions for the partition $(2, 1)$ are given by $(i, j, k), (i, k, j), (k, i, j)$. The permutation of i and j yields the same term, as they originate from $R^{(2)}$. Replacing the labels i, j, and k with the corresponding order of the term, the permutations become $(2, 2, 1), (2, 1, 2), (1, 2, 2)$. These are just the unique permutations of $(2, 2, 1)$. As the states in any direct product basis may be labeled by unique permutations of integer partitions, libraries for the fast evaluation of unique permutations already exist, and we may reuse them in our computation of $T(p)$, with slight modifications.

To make this process clearer, we will consider the partition $(2, 2, 1)$, corresponding to contractions of the form $[R^{(2)}R^{(2)}R^{(1)}V^{(3)}]$ which arise in the evaluation of 5th order derivatives. First, we will introduce the cross-block indexing vector where each partition symbol is repeated a number of times equal to its value, e.g.

$$I_C(2, 2, 1) = (2, 2, 2, 2, 1)$$

This represents the set of unique values in the corresponding $R^{(k)}$ tensors. We first compute the five unique permutations of this set. Then we introduce intra-block indexing vectors to account for the permutations necessary between repeated derivative tensors at the same order

$$I_B(2, 2) = (2, 2, 1, 1)$$

$$I_B(1) = (1)$$

Here we compute the unique permutations under a second condition, that blocks of terms cannot "switch", that is, $(2,1,2,1)$ is a valid permutation, but $(1,2,1,2)$ is not. It is sufficient in this case to simply check that the set of first positions of a given entry (2 or 1 in this case) is ordered. This process may be implemented efficiently, and the storage and precomputation of $T(p)$ is possible for repeated use.

S3. INTERPOLATING BETWEEN COORDINATE SYSTEMS

We describe here a simple interpolation using the transformations described here that can be extended into more sophisticated schemes. Given an initial Cartesian configuration of a system X_0 with corresponding internal coordinate system R_0 and a final configuration X_1 with corresponding coordinate system R_1, we will define our interpolated configurations as

$$X(p) = f^{-1}(f(X_0; R_0)(1 - w(p)) + f(X_1; R_1)w(p); R) \tag{S6}$$

where $p \in [0, 1]$, f is the conversion to a common coordinate coordinate system, R, which may be either R_0, R_1, or a wholly different coordinate system, such as a reduced set of interatomic distances, and $w(p)$ is a weighting function. For a simple linear interpolation, $w(p) = p$, however we have found it effective to use a weighting function based on the displacement of each structure under a simpler interpolation model.

In particular, letting

$$n_0 = |f(X_0; R_0) - f(X_1; R_0)| \tag{S7}$$

$$n_1 = |f(X_1; R_1) - f(X_0; R_1)| \tag{S8}$$

we can define the cross-over point where interpolating in the initial coordinate system involves larger distortions (in local coordinate space) than displacement in the final coordinate system as $c = n_1/(n_0 + n_1)$ and choose a sigmoid to be centered at this point by letting

$$w(p) = \frac{1}{1 + \exp(-k(c - p))} \tag{S9}$$

where k controls the steepness of the switching. To ensure a proper interpolation, k should be chosen such that $w(0) \approx 1$ and $w(1) \approx 0$. To obtain this behavior, we let

$$k = \begin{cases} -\frac{20}{1-c} & c > .5 \\ -\frac{20}{c} & c < .5 \end{cases} \tag{S10}$$

which ensures that independent of c the largest possible deviation is less than 10^{-8}. When R_1 and R_2 consist of different numbers of distances and angles, extra care must be taken to ensure n_0 and n_1 are comparable and c is unitless. Other norms that measure the distortion within a local coordinate system may, of course, be used as well.

It should be noted that this is just one choice of interpolant, and other forms may easily be used once it is straightforward to convert between coordinate systems. As an example, one could also consider an exponentially decaying weighting function given by

$$w(p) = 1 - \exp\left(-\frac{n_1}{n_0}\frac{p}{1-p}\right) \tag{S11}$$

which can be be derived by taking exponential of the ratio of the linear interpolation of X_0 and X_1 in their respective coordinate systems. This form has the benefit of accounting for the difference in n_0 and n_1 while enforcing $w(0) = 1$ and $w(1) = 0$, but only slowly switches from R_0-like to R_1-like.

S4. INTERPOLATION ENERGIES USING MLIPS

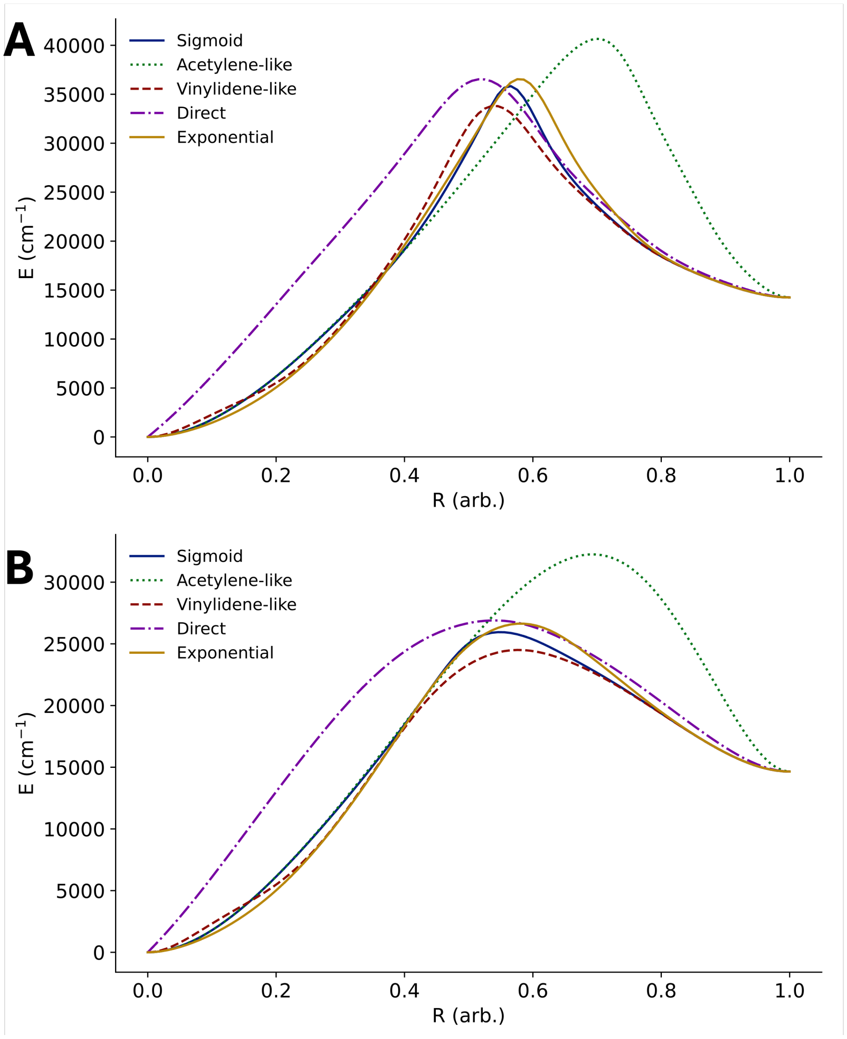

FIG. S1. (A) Comparison of energies of the ground state using AIMNet2 and the coordinate system interpolations as described in the text (B) Comparison of energies of the ground state using MACE and the coordinate system interpolations as described in the text

S5. NORMAL MODES OF CYCLOPENTADIENYL MOLYBDENUMTRICARBONYL

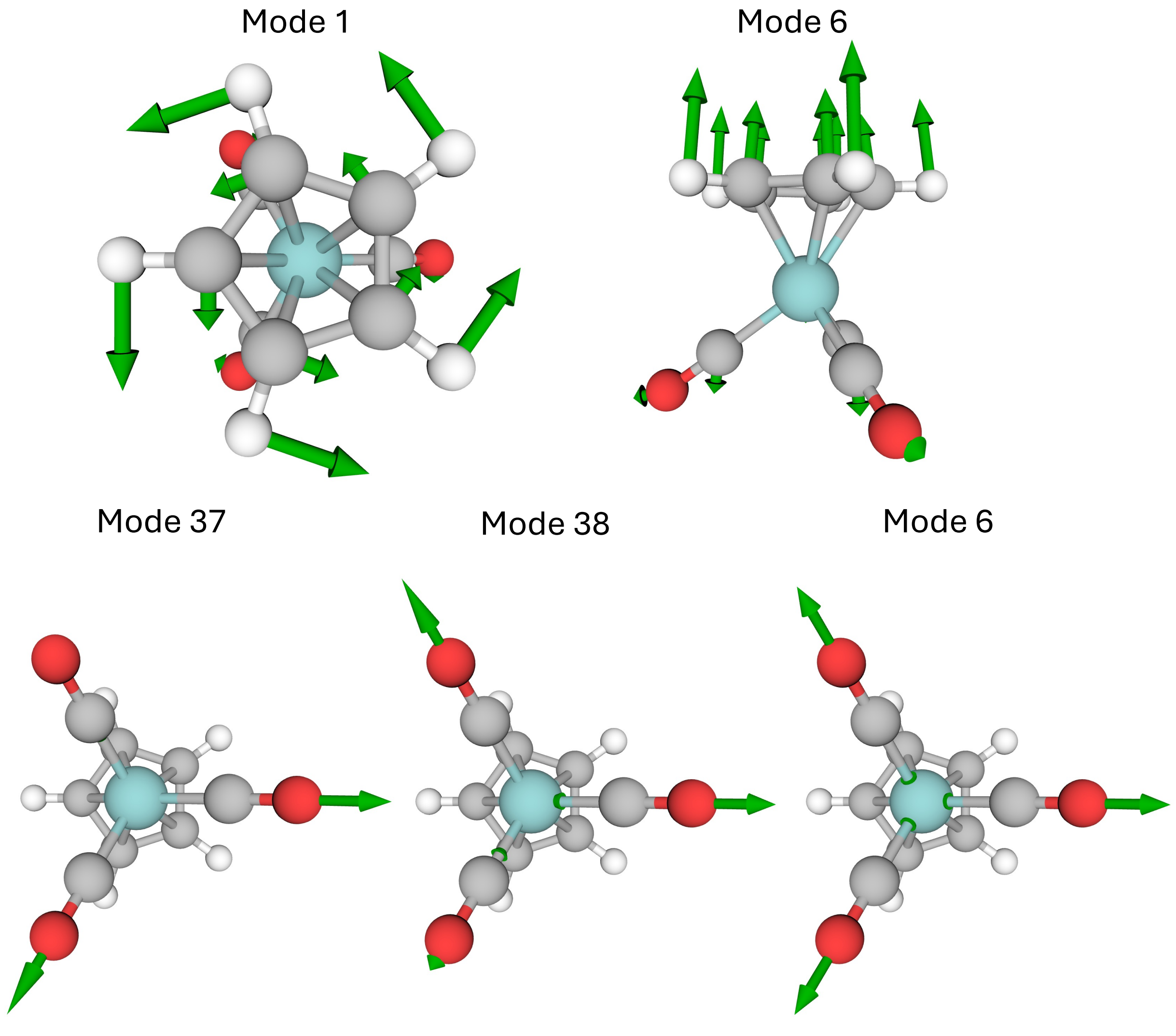

FIG. S2. Subset of the normal modes for $[CpMo(CO)_3]^{-1}$, depicted are the lowest frequency cyclopentadienyl torsion, the complex stretch, and the asymmetric CO stretches, and the symmetric CO stretch.

S6. NORMAL MODES OF METHANOL

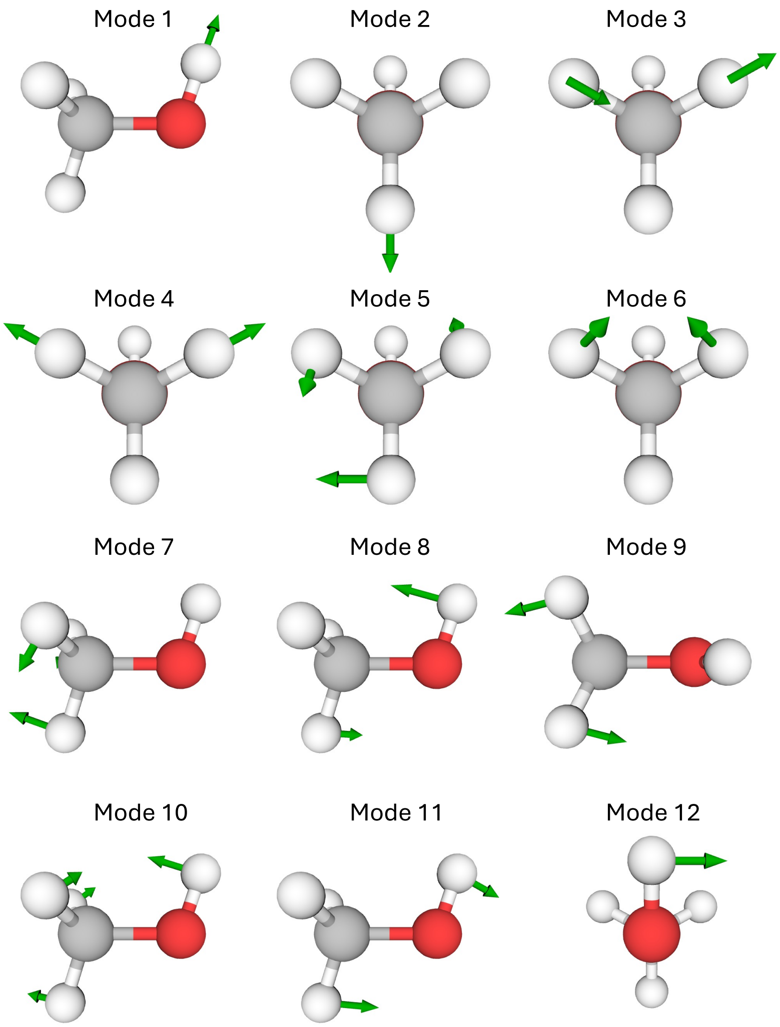

FIG. S3. Normal modes for methanol, depicted are the OH stretch, the *anti*-CH stretch, the asymmetric stretch of the *gauche*-CH groups, the *gauche*-symmetric stretch, the *gauche*-CH wag with *anti*-CH torsion, the *gauche*-CH bend, the total CH bend, the out-of-phase COH bend with *anti*-HCO bend, the pure *gauche*-CH wag, the COH bend with total methyl rock, the in-phase COH bend with *anti*-HCO bend, and the OH torsion

S7. TABLE OF ANHARMONIC FREQUENCIES

	B3LYP		wB97X-D3		AIMNet	
States	Cart.	Int.	Cart.	Int.	Cart.	Int.
OH	3647	3647	3708	3708	3673	3673
CH	2972	2972	2970	2970	2961	2961
CH	2913	2913	2881	2881	2979	2979
CH	2714	2714	3207	3207	2920	2920
HCH	1466	1466	1474	1474	1524	1524
HCH	1458	1458	1471	1471	1516	1516
HCH	1446	1446	1455	1455	1509	1509
HOCH	222	223	11	12	72	75

TABLE S1. Internal vs cartesian comparison without reaction path projection and no degeneracy handling. Identical up to numerical stability issues.

S8. TABLE OF ANHARMONIC FREQUENCIES USING MLIP LEVELS OF THEORY

	AIMNet2				MACE			
	0		-60		0		-60	
States	Harm.	Anh.	Harm.	Anh.	Harm.	Anh.	Harm.	Anh.
OH	3917	3674	3906	3477	3856	3687	3885	3719
CH	3135	2962	3096	3000	3139	2989	3111	2870
CH	3076	2979	3089	2965	3076	2862	3102	2850
CH	3030	2881	3030	2878	3025	2808	3040	2778
HCH	1513	1524	1504	1541	1502	1460	1509	1465
HCH	1480	1514	1476	1508	1491	1454	1481	1442
HCH	1473	1509	1474	1502	1472	1442	1477	1446
HCCH	206	70	-197	-462	280	234	-285	-436

TABLE S2.

S9. TABLE OF ANHARMONIC FREQUENCIES USING DFT LEVELS OF THEORY

	B3LYP				wB97			
	0		-60		0		-60	
States	Harm.	Anh.	Harm.	Anh.	Harm.	Anh.	Harm.	Anh.
OH	3829	3647	3864	3516	3918	3708	3952	3572
CH	3108	2951	3077	2928	3134	2951	3103	2896
CH	3040	2896	3067	2924	3067	2874	3094	2908
CH	2994	2848	3011	2867	3013	2816	3028	2836
HCH	1509	1466	1516	1471	1516	1474	1523	1481
HCH	1498	1458	1489	1447	1506	1471	1496	1454
HCH	1477	1446	1481	1449	1488	1455	1492	1457
HCCH	291	222	-293	-451	298	11	-292	-652

TABLE S3.

S10. HARMONIC FREQUENCY COMPARISON OF CH STRETCHES IN METHANOL

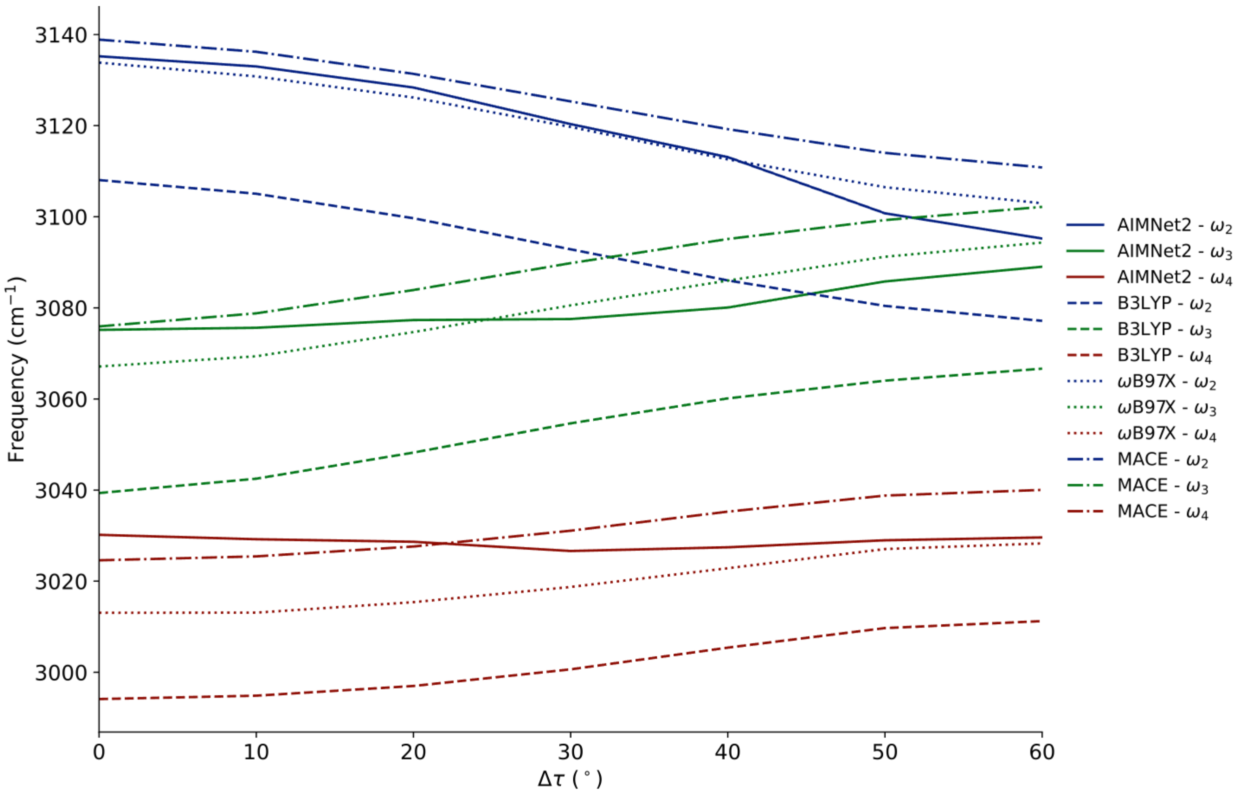

FIG. S4. Harmonic frequency of the CH stretches in methanol evaluated at different distortion angles

S11. HARMONIC FREQUENCY COMPARISON OF THE OH STRETCH IN METHANOL

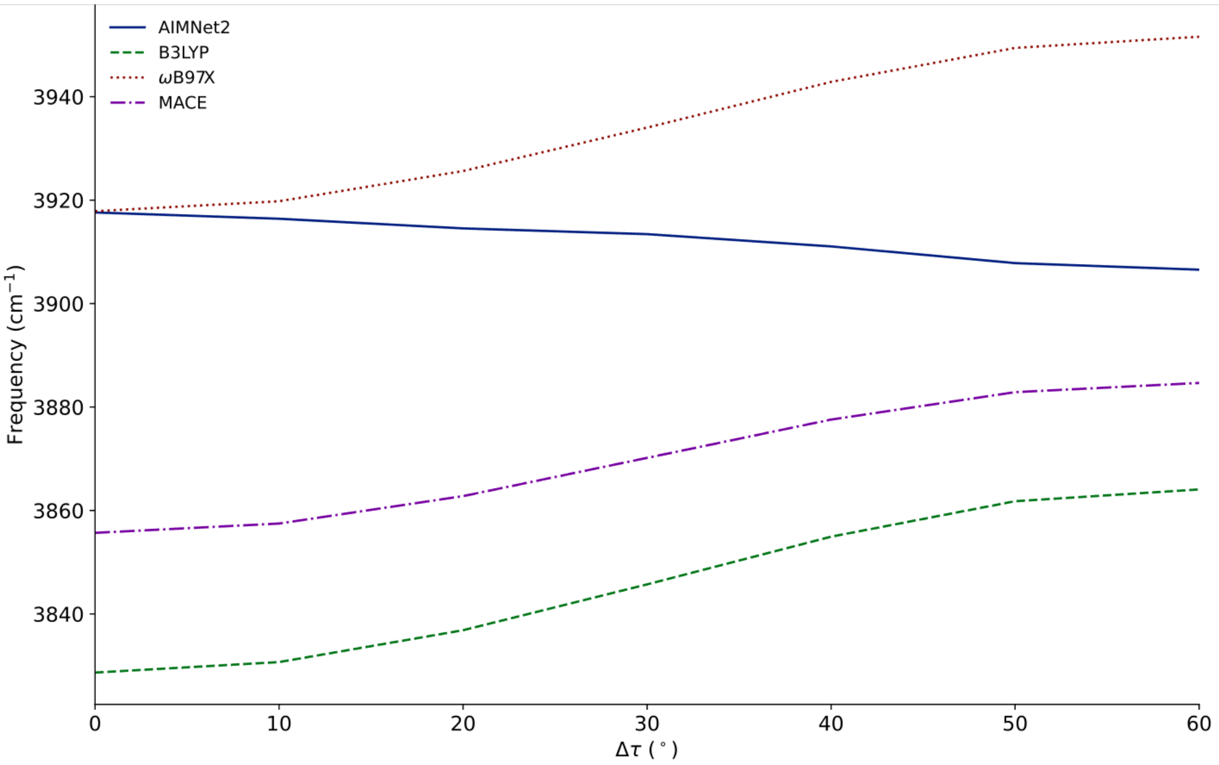

FIG. S5. Harmonic frequency of the OH stretch in methanol evaluated at different distortion angles

S12. ANHARMONIC FREQUENCY COMPARISON OF THE OH STRETCH IN METHANOL

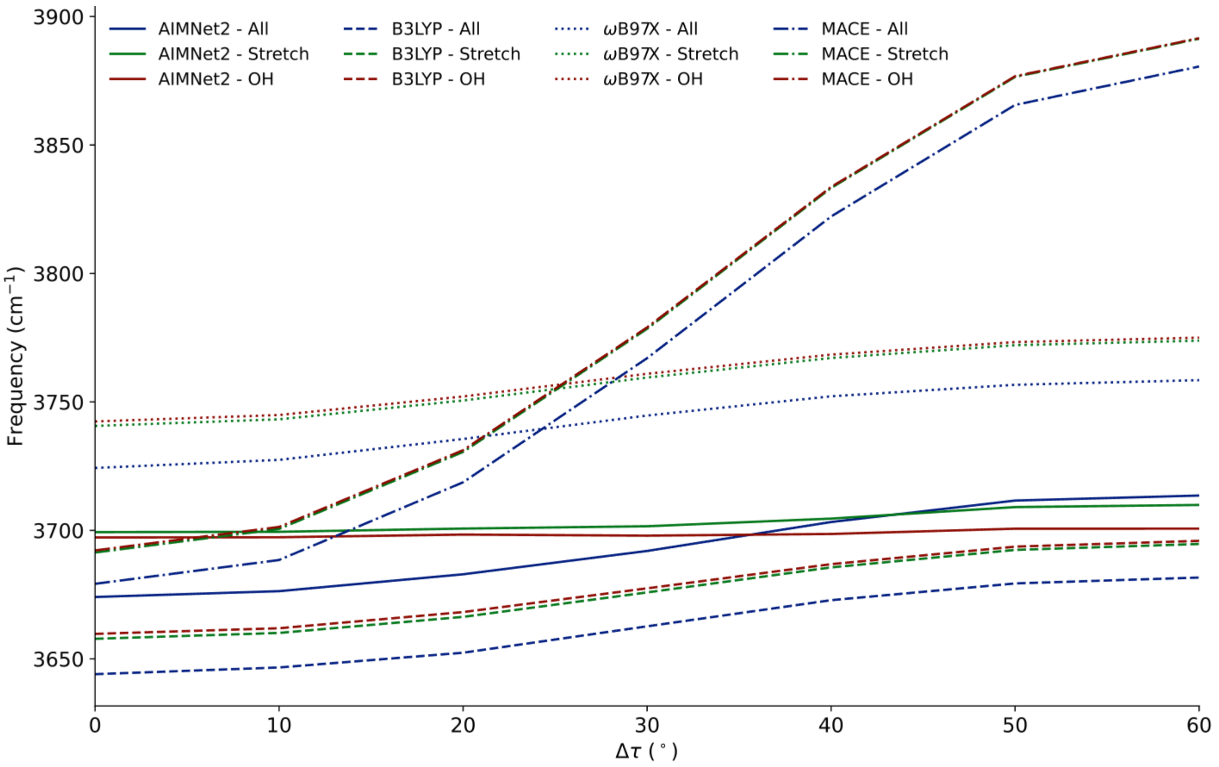

FIG. S6. Anharmonic frequency of the OH stretch in methanol evaluated at different distortion angles and different subspaces

S13. PURE ANHARMONICITY OF THE OH STRETCH IN METHANOL

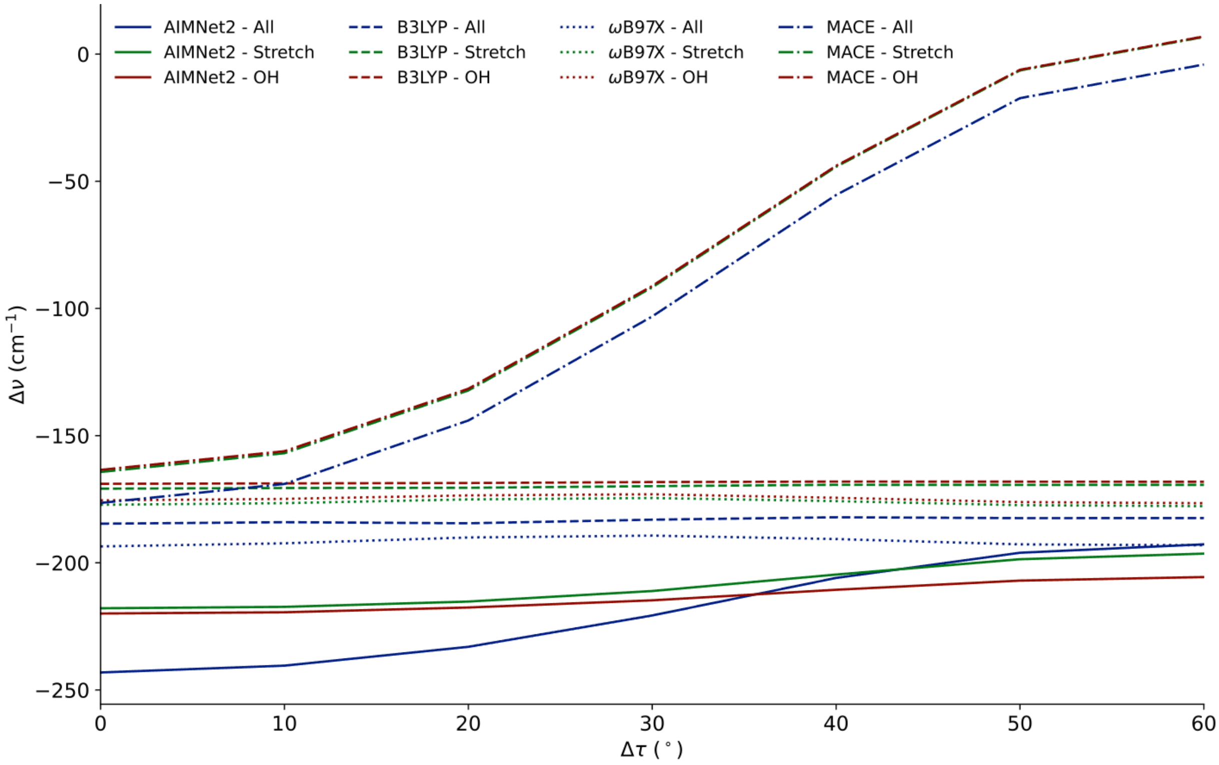

FIG. S7. Comparison of pure anharmonic contributions to the OH stretch in methanol across subspaces and levels of theory

REFERENCES

[1]G. J. Small, The Journal of Chemical Physics **54**, 3300–3306 (1971).
